# Antibodies targeting a shared epitope exploit IgE allostery to drive distinct functional outcomes

**DOI:** 10.1073/pnas.2613091123

**Published:** 2026-09-08

**Authors:** Anna M. Davies, Nyssa Drinkwater, Andrew J. Beavil, Victoria O’Dowd, Daniel Lightwood, Tom Ceska, Alistair J. Henry, Brian J. Sutton, James M. McDonnell

**Affiliations:** ^a^ Randall Centre for Cell and Molecular Biophysics, King’s College London, New Hunt’s House, London SE1 1UL, United Kingdom; ^b^UCB, Slough SL1 3WE, United Kingdom

**Keywords:** immunoglobulin E, antibody discovery, allosteric modulation, conformational plasticity, structural immunology

## Abstract

The Fc region of immunoglobulin E (IgE-Fc) is an important therapeutic target due to the antibody’s critical role in allergic disorders through interactions with high-affinity (FcεRI) and low-affinity (CD23) receptors. IgE-Fc is known to be conformationally flexible, undergoing large-scale structural changes that modulate receptor binding and downstream biological functions. The mechanistic basis for how ligands induce these allosteric changes is not fully understood. In this study, we investigate structure–function relationships of IgE by characterizing four closely related anti-IgE-Fc Fab fragments with highly conserved sequences that recognize a shared epitope at the base of the Cε2 domain. Despite their sequence similarity, these Fabs exhibit markedly different binding characteristics, stoichiometries, and functional activities. Furthermore, the crystal structures of these four Fab complexes reveal that the bound IgE-Fc adopts a wide range of conformations. These structural differences directly dictate the functional outcomes, ranging from conformations that facilitate FcεRI binding to those that prevent or destabilize receptor interaction. Our findings demonstrate that highly similar antibodies binding to the same epitope on a protein that exhibits significant conformational plasticity can elicit distinct and divergent functional outcomes. This work challenges the conventional antibody discovery paradigm, i.e., “same epitope, same functional outcome,” and highlights the importance of accounting for both the target’s structural flexibility and the resulting functional diversity within antibody selection strategies. This is especially critical when targeting dynamic proteins where allosteric mechanisms are fundamental to function.

## Significance

Antibody discovery typically operates on the “same epitope, same function” paradigm. However, for dynamic proteins like immunoglobulin E (IgE), the primary mediator of allergic disease, this approach is overly simplistic. By characterizing four closely related antibodies that recognize the same epitope but induce divergent functional outcomes, we demonstrate that antibodies can act as molecular “switches” to capture distinct conformational states of IgE-Fc. We show that even a single amino acid difference can dictate whether an antibody stabilizes a “bent” or “extended” conformation, thereby controlling inhibition or enhancement of receptor binding. This study provides a mechanistic framework for understanding how allosteric transitions modulate antibody function and suggests that prioritizing conformational capture over epitope diversity is essential for engineering next-generation therapeutics.

The structure of immunoglobulin E (IgE) is of considerable interest due to the key role played by this antibody in allergic disorders (1, 2). The Fc region of IgE (IgE-Fc), which is responsible for receptor interactions, comprises two identical chains of Cε2, Cε3, and Cε4 domains. IgE-Fc binds two principal receptors, FcεRI and CD23 (2, 3). The high affinity interaction between IgE-Fc and FcεRI, responsible for mast cell and basophil degranulation, is a long-standing target for therapeutic intervention (4). The interaction with CD23, which plays a role in diverse processes such as regulation of IgE synthesis, antigen presentation, and transcytosis of IgE-immune complexes (1, 5–7), is also a potentially promising target in the treatment of allergic disease (8).

Three-dimensional structures have been determined for IgE-Fc and subfragments of IgE-Fc (9–13), complexes with soluble FcεRI and CD23 (14–20), and complexes with a variety of anti-IgE molecules, including the clinically approved omalizumab (8, 21–29). In these structures, the bend in IgE-Fc, which is defined as the angle between the local two-fold axes of the (Cε2)_2_ and (Cε4)_2_ domain pairs (9), ranges from ~54° (acutely bent) to ~180° (fully extended) (8–10, 14, 15, 21, 22, 27, 29) (*SI Appendix*, Fig. S1).

The Fc region of IgE is thus capable of undergoing large-scale conformational changes, and this conformational flexibility plays an important role in the different biological functions of IgE (21). An allosteric communication pathway that prevents simultaneous engagement of CD23 and FcεRI receptors has been identified (16, 30). The Cε3 domains can adopt a variety of positions relative to the (Cε4)_2_ domain pair, and adopt “open” and “closed” conformations respectively when in complex with soluble FcεRI and CD23 (14, 16). Furthermore, it has been shown that the Cε3 domains within IgE-Fc that bind to both cellular receptors possess an unusual conformational plasticity (31, 32). Of the various allosterically mediated processes observed for IgE-Fc, the phenomenon of induced dissociation is particularly noteworthy (2) and is dependent on IgE’s unusual conformational dynamics and its consequent capacity for allosteric modulation.

In this study, we report a set of four anti-IgE-Fc antibodies with remarkably similar sequences. Although these antibodies share a common binding epitope, the structures of these four IgE-Fc/Fab complexes are distinctly different, particularly with regard to the conformation of the IgE-Fc, and the antibodies have markedly different functional properties. The mechanistic basis for these structural and functional differences is investigated and described herein.

## Results

### Derivation and Sequence Comparison of Four Anti-IgE-Fc Fabs.

As part of ongoing efforts to elucidate structure/function relationships for IgE antibodies, we undertook a program of work to identify and characterize a panel of antibodies against the Fc region of IgE. These antibodies were derived using previously described methods, in which antibody variable regions are extracted from individual antigen-specific B cells (33, 34). Experiments were performed to measure binding affinities, identify binding epitopes, and characterize functional activities of these antibodies; results from these experiments are described below. Four closely related antibodies are described in this study. In the traditional antibody sequence–structure–function paradigm, antibodies with very similar sequences in their complementarity-determining regions (CDRs) may be anticipated to bind to the same epitope on the target antigen, and so would be expected to have similar functional activities. The anti-IgE-Fc antibodies described in this work are noteworthy for sharing a similar epitope but having markedly different activities.

**Fig. 1.**
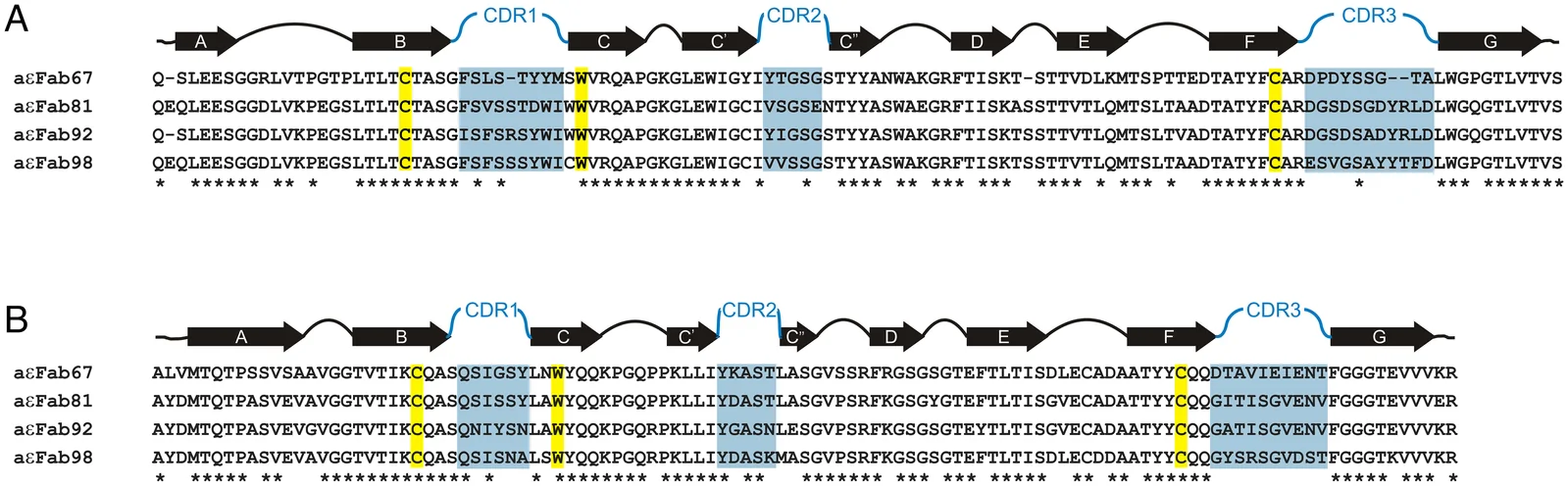
A comparison of the sequences of four anti-IgE-Fc Fabs. (*A*) Alignment of heavy chain variable domains, and (*B*) alignment of light chain variable domains. Approximate locations of β-strands are indicated by cartoons above the sequences. Residues of the conserved “central pin” (35) of Ig-domains are highlighted in yellow, and CDR residues are highlighted in blue. Where all four Fabs show identical amino acids in the aligned sequences, this is indicated with an asterisk below the sequence. The closest putative variable (V) and joining (J) gene families were assigned via NCBI IgBLAST against the *Oryctolagus cuniculus* database. Heavy chain V_H_ and light chain V_L_ germline family assignments are as follows: aεFab67: IGHV1S69, IGHJ2, IGKV1S34, IGKJ1-2; aεFab81: IGHV1S45, IGHJ3, IGKV1S3, IGKJ1-2; aεFab92: IGHV1S45, IGHJ3, IGKV1S37, IGKJ1-2; aεFab98: IGHV1S45, IGHJ2, IGKV1S3, IGKJ1-2. For the light chains, the closest germline database hits for V1S3, V1S34, and V1S37 displayed high sequence parity (differing by fewer than five nucleotides). Due to this intrinsic germline duplication and the confounding effects of somatic hypermutation, these assignments represent the top matching family hits rather than definitive single-gene resolution. Due to the short length of rabbit diversity (D) segments and low mathematical confidence scores across both IMGT and IgBLAST databases, heavy chain D gene assignments were undetermined.

The four anti-epsilon chain antibodies were produced as Fab fragments and are called aεFab67, aεFab81, aεFab92, and aεFab98. The amino acid sequences of their heavy- and light-chain variable domains are shown in Fig. 1. Although each sequence was derived from a separate isolated B cell, their high sequence similarity suggests they may share a common ancestral lineage. These four antibodies utilize closely related V_H_ and V_L_ gene families (Fig. 1). The four utilize the similar V_H_ and V_L_ gene families (Fig. 1). Outside of the CDRs, the average pairwise sequence identities for these four antibodies are >88%, for both light chain and heavy chain domains. Among these four related antibodies, aεFab81 and aεFab92 are most similar to each other; for example, in both heavy and light chains their CDR3 sequences differ by only a single amino acid (Fig. 1). aεFab81 and aεFab67 are the least similar; but even in these most dissimilar antibodies, their CDRL1 and CDRL2 sequences differ only by a single amino acid each (Fig. 1). Despite the high sequence similarities of these antibodies (and their presumed sharing of at least highly overlapping epitopes, as indeed we confirm below), these antibodies engage IgE-Fc differently and display marked differences in functional activities.

### Four Closely Related Anti-IgE-Fc Fabs Have Markedly Different Binding Characteristics.

**Fig. 2.**
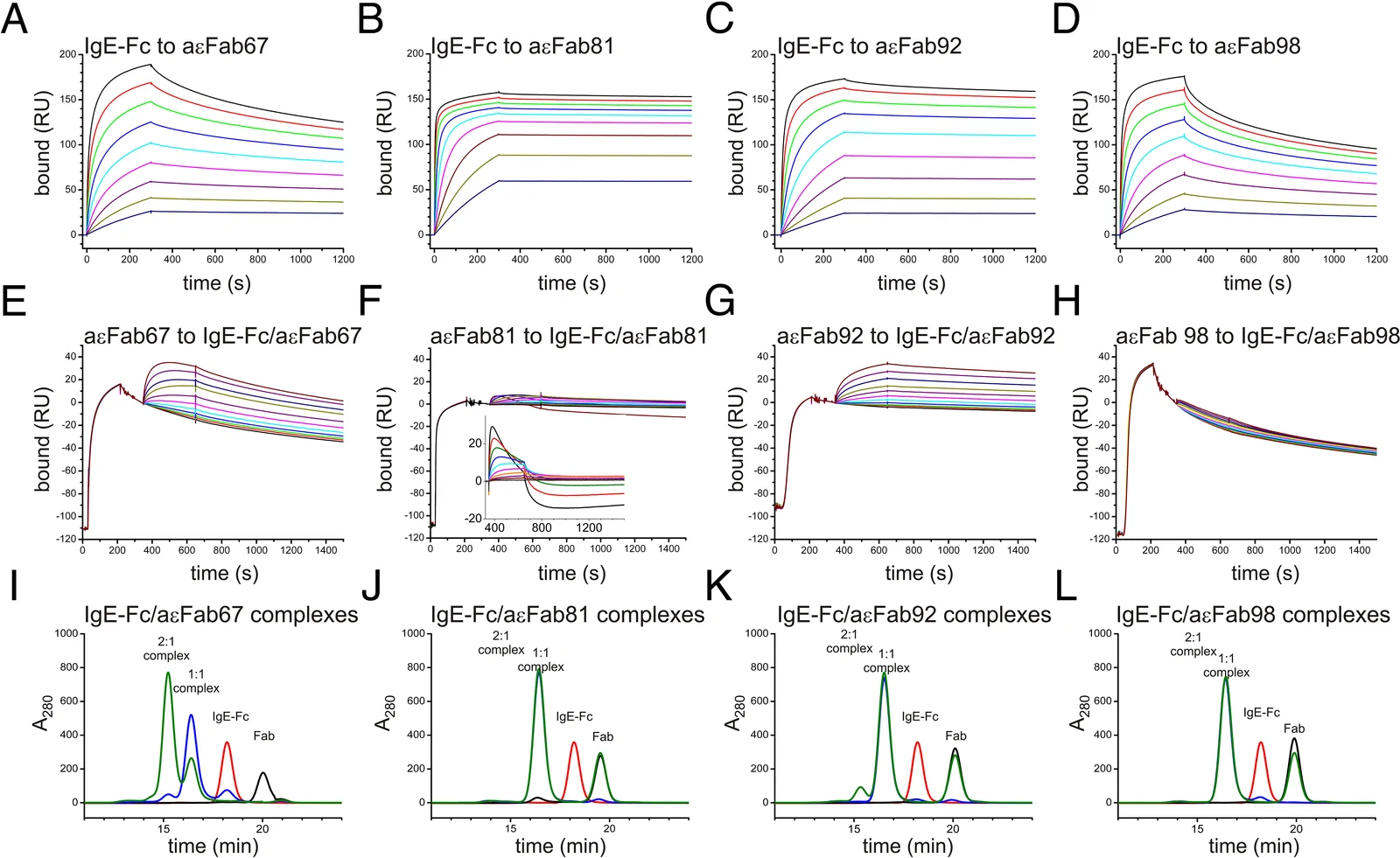
Affinities and stoichiometries of four anti-IgE-Fc Fabs. Direct binding of IgE-Fc to immobilized aεFab67 (*A*), aεFab81 (*B*), aεFab92 (*C*), and aεFab98 (*D*). Binding of a second Fab to preformed IgE-Fc/Fab complexes (*E*–*H*). The *Inset* in (*F*) highlights the induced dissociation of the IgE-Fc–aεFab81 complex by the second aεFab81 molecule. For direct binding experiments a twofold dilution series was performed with a highest concentration of 100 nM. For the binding of the second Fab, the highest concentration used was 250 nM. Size-exclusion chromatography was performed on anti-IgE-Fc Fab/IgE-Fc complexes (*I*–*L*). Complexes were set up at 0:1 (red), 1:1 (blue), and 2:1 (green) molar ratios (anti-IgE-Fc Fab:IgE-Fc) to assess the stoichiometries of complexes.

The four Fabs were initially assayed for direct binding to IgE-Fc. All four Fabs showed high-affinity binding to IgE-Fc, with affinities ranging from about 15 pM to about 380 pM (Fig. 2 *A*–*D* and *SI Appendix*, Table S1). It is not unexpected that small differences in antibody sequences can lead to substantial changes in antibody affinities for their targets; this has been described and characterized in many systems (e.g., refs. 36–38). The surface plasmon resonance (SPR) binding curves for two Fabs, aεFab67 (Fig. 2*A*) and aεFab98 (Fig. 2*D*), were clearly biphasic (*SI Appendix*, Fig. S2), a characteristic that has been observed for other anti-IgE-Fc antibodies (2, 21, 27). This can arise from the dimeric nature of IgE-Fc. Although IgE-Fc is a homodimer, as are all immunoglobulin Fc regions, the dimer can be structurally asymmetric (9, 10, 14, 15, 27). Consequently, some anti-IgE-Fc antibodies bind to epitopes that are symmetrically related, one in each chain, while some bind to asymmetrically disposed epitopes, the latter leading to biphasic binding kinetics. Although a 2:1 binding stoichiometry might be expected for binding to the homodimeric IgE-Fc, some anti-IgE-Fc antibodies may simply bind with a 1:1 stoichiometry as does, for example, sFcεRIα (the extracellular IgE-binding domains of the α-chain) (14, 19, 39, 40); monophasic binding does not therefore indicate symmetrical 2:1 binding to the homodimer.

To determine binding stoichiometries, we tested the ability of each of the Fabs to bind to a Fab/IgE-Fc complex in an SPR sandwich experiment, and also used size-exclusion chromatography (SEC) to identify 1:1 or 2:1 Fab/IgE-Fc complexes. In SPR binding experiments, all four Fabs showed some capacity to form a 2:1 complex but to markedly different degrees (Fig. 2 *E*–*H*). aεFab67 readily formed a 2:1 complex (Fig. 2 *E* and *I*). aεFab92 formed a 2:1 complex, but it was less stable than the aεFab67 2:1 complex in the SEC experiment (Fig. 2 *G* and *K*). aεFab98 showed very little capacity to form a 2:1 complex (Fig. 2 *H* and *L*) and aεFab81 virtually none at all (Fig. 2 *F* and *J*). At very high concentrations aεFab81 could be forced to form a 2:1 complex, but the binding of the second aεFab81 destabilized the existing 1:1 aεFab81/IgE-Fc complex (Fig. 2 *F*, *Inset*). This is a form of allosterically induced dissociation, a phenomenon that has been observed in multiple IgE-Fc/ligand interactions (2, 27). Given the similarities of the four Fab sequences, and the presumed similarity of the epitopes that they recognized, it was very surprising to observe such substantial differences in their binding characteristics and stoichiometries.

**Fig. 3.**
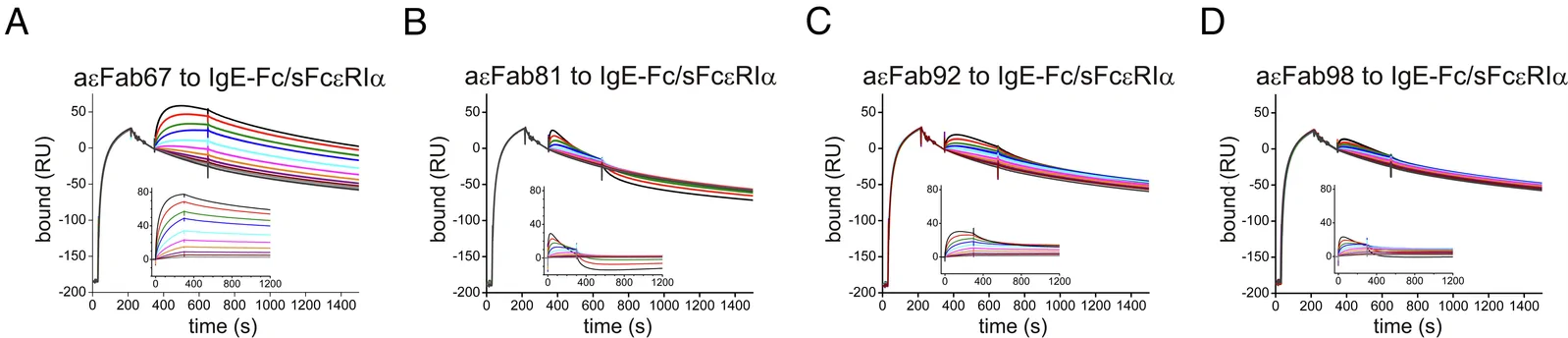
Anti-IgE-Fc Fab binding to the IgE-Fc/sFcεRIα complex. sFcεRIα was immobilized on a CM5 chip, and IgE-Fc was captured at a concentration of 50 nM. Twofold concentration series of aεFab67 (*A*), aεFab81 (*B*), aεFab92 (*C*), and aεFab98 (*D*), starting at a concentration of 250 nM, were flowed over the IgE-Fc–sFcεRIα complex. Insets show baseline subtracted binding curves.

The four Fabs also differentially affect and are affected by the binding of the IgE receptor sFcεRIα. aεFab67 bound tightly to an IgE-Fc/sFcεRIα complex (Fig. 3*A*), and binding was consistent with a 1:1:1 aεFab67/IgE-Fc/sFcεRIα complex. The three other Fabs bound markedly less well to the IgE-Fc/sFcεRIα complex (Fig. 3 *B*–*D*) and, to varying degrees, destabilized the IgE-Fc/sFcεRIα complex. aεFab81 showed the greatest capacity for induced dissociation of IgE-Fc from sFcεRIα (Fig. 3 *B*, *Inset*), aεFab98 to a lesser degree (Fig. 3 *D*, *Inset*), and aεFab92 to a barely detectable level (Fig. 3 *C*, *Inset*). While there are differing degrees of competition occurring between sFcεRIα and three of the anti-IgE-Fc Fabs (aεFab81, aεFab98, and aεFab92), aεFab67 and sFcεRIα show positive cooperativity in their binding. In a striking side-by-side comparison, sFcεRIα has an association rate more than five times faster for an IgE-Fc/aεFab67 complex compared to unbound IgE-Fc (*SI Appendix*, Fig. S3). Insights into the reasons for these strikingly different functional differences, derived from the three-dimensional structures of all four Fab/IgE-Fc complexes, are discussed below.

### Overall Structures of the aεFab/IgE-Fc Complexes.

**Fig. 4.**
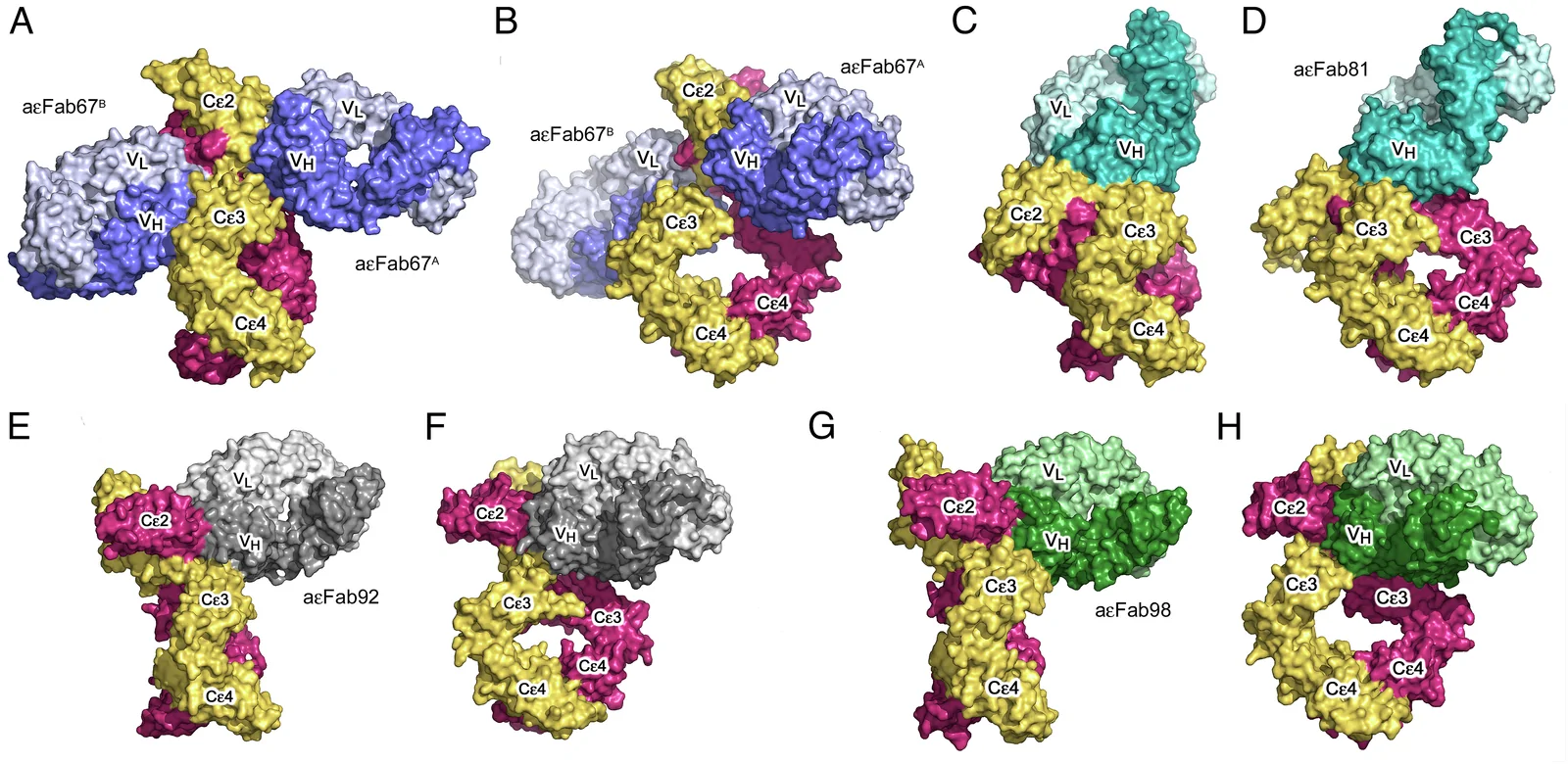
Overall structures of the aεFab/IgE-Fc complexes. (*A*) aεFab67 (dark blue and light blue) binds IgE-Fc with 2:1 stoichiometry. IgE-Fc adopts a partially extended conformation. (*B*) View of the aεFab67/IgE-Fc complex from the front face of the Fcε3-4 region. (*C*) aεFab81 (dark cyan and pale cyan) binds IgE-Fc with 1:1 stoichiometry. IgE-Fc adopts an acutely bent conformation. (*D*) View of the aεFab81/IgE-Fc complex from the front face of the Fcε3-4 region. (*E*) aεFab92 (dark gray and light gray) binds IgE-Fc with 1:1 stoichiometry. IgE-Fc adopts a partially extended conformation. (*F*) View of the aεFab92/IgE-Fc complex from the front face of the Fcε3-4 region. (*G*) aεFab98 (dark green and pale green) binds IgE-Fc with 1:1 stoichiometry. IgE-Fc adopts a partially extended conformation. (*H*) View of the aεFab98/IgE-Fc complex from the front face of the Fcε3-4 region. In panels *A*–*H*, IgE-Fc chains A and B are colored yellow and pink, respectively.

We solved crystal structures of the four aεFabs in complex with IgE-Fc (Fig. 4). The 2:1 aεFab67/IgE-Fc complex (Fig. 4 *A* and *B*) and 1:1 aεFab81/IgE-Fc complex (Fig. 4 *C* and *D*) were solved at 3.2 Å resolution. The 1:1 aεFab92/IgE-Fc (Fig. 4 *E* and *F*) and aεFab98/IgE-Fc (Fig. 4 *G* and *H*) complexes were solved at 3.5 Å and 2.4 Å resolution, respectively. IgE-Fc adopts strikingly different conformations in these complexes, ranging from acutely bent to partially extended, which is discussed in more detail later.

The structures reveal that the aεFabs do indeed share a common epitope, but engage different Cε2 domains: aεFab67^A^ and aεFab67^B^ (Fig. 4*A*) bind the Cε2 domains in chains A and B, respectively, aεFab81 (Fig. 4*C*) binds the Cε2 domain in chain A, while aεFab92 (Fig. 4*E*) and aεFab98 (Fig. 4*G*) bind the Cε2 domain in chain B. For consistency, the chain assignments in IgE-Fc are the same as those established when the crystal structure of the complete Fc region was first solved (9). In this acutely bent structure, the (Cε2)_2_ domain pair contacts the Cε3 domain in chain A. The chain assignments in the partially extended conformations reported here are such that the (Cε2)_2_ domain pair remains on the same side of the IgE-Fc region as in the acutely bent structure.

### The Four aεFabs Share a Common Epitope.

**Fig. 5.**
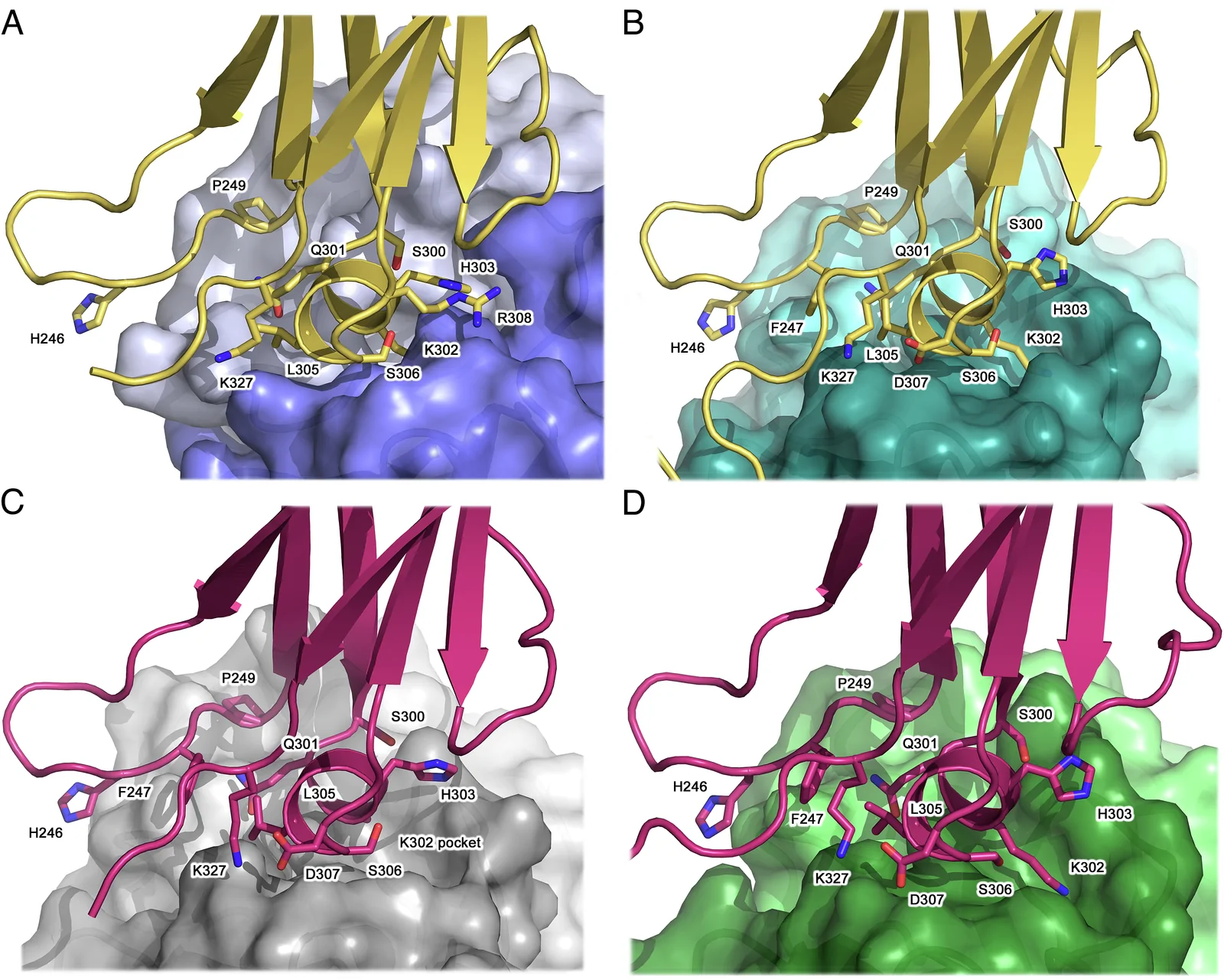
Four different aεFabs bind a common epitope. (*A*) Interface between aεFab67^A^ (V_H_ dark blue and V_L_ light blue) and the Cε2 domain in chain A (yellow). (*B*) Interface between aεFab81 (V_H_ dark cyan and V_L_ pale cyan) and the Cε2 domain in chain A (yellow). (*C*) Interface between aεFab92 (V_H_ dark gray and V_L_ light gray) and the Cε2 domain in chain B (pink). (*D*) Interface between aεFab98 (V_H_ dark green and V_L_ pale green) and the Cε2 domain in chain B (pink). The Fabs recognize a common epitope at the base of the Cε2 domain, which includes H246, F247, P249, T250, S300-H303, L305-R308, and K327. The K302 side chain is partially disordered in the aεFab92/IgE-Fc complex (*C*), but the location of the K302 pocket is indicated.

**Fig. 6.**
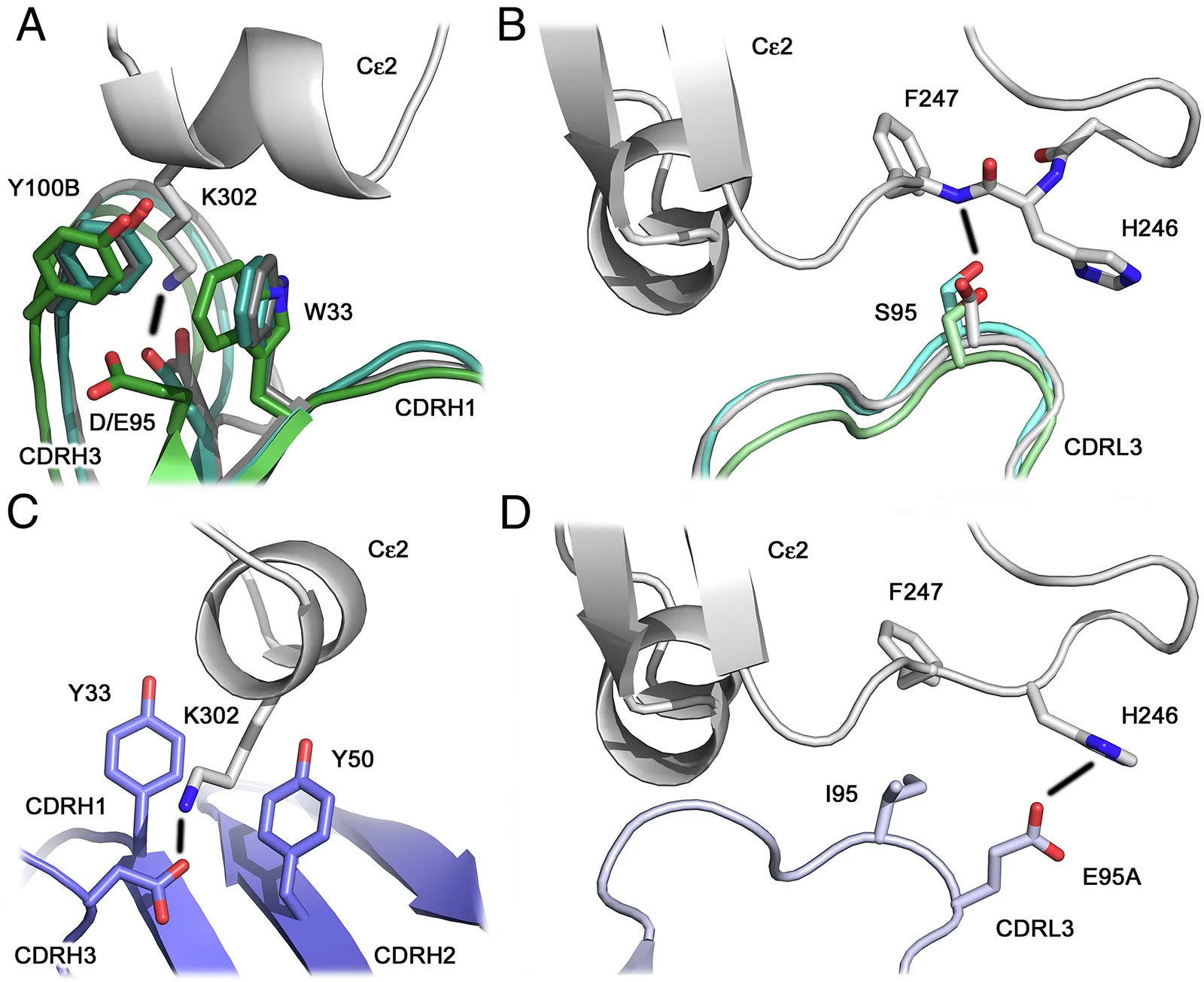
Features of the interface with the common epitope. (*A*) In the complexes with aεFab81 (dark cyan), aεFab92 (dark gray) and aεFab98 (dark green), W33 and Y100B form one side of the K302 binding pocket and D/E95 lines its base. K302 forms a hydrogen bond with D95 and a salt bridge with E95. (*B*) In the complexes with aεFab81 (pale cyan), aεFab92 (gray) and aεFab98 (pale green), H246 and F247 (Cε2) pack against CDRL3, with a hydrogen bond between F247 and S95 (CDRL3). (*C*) In the complex with aεFab67 (dark blue), Y33 and Y50 form one side of the K302 binding pocket and D95 lines its base. K302 forms a hydrogen bond with D95. (*D*) In the complex with aεFab67 (light blue), H246 and F247 (Cε2) contact CDRL3, with a salt bridge between H246 and E95A (CDRL3). In panels *A*–*D*, aεFab residues are numbered according to the Chothia numbering system. In panels *A*–*D*, the Cε2 domain is colored light gray. Hydrogen bonds and salt bridges are depicted as black lines.

The four aεFabs share an epitope at the base of the Cε2 domain (Fig. 5), burying an average surface area of ~660 Å^2^. The common epitope comprises His246, Phe247, Pro249, and Thr250 from the loop between β-strands A and B, Ser300-His303 and Leu305-Arg308 from the loop and short α helix between β-strands E and F, and Lys327 from the loop region that follows β-strand G. A key feature in all four complexes is binding of Lys302 (Cε2 domain) in a pocket created by the aεFab (Figs. 5 and 6). As mentioned above, aεFab67^A^ and aεFab67^B^ bind the Cε2 domains in chains A and B, respectively (Figs. 4*A* and 5*A*), aεFab81 binds the Cε2 domain in chain A (Figs. 4*C* and 5*B*), while aεFab92 and aεFab98 bind the Cε2 domain in chain B (Figs. 4 *E* and *G* and 5 *C* and *D* and *SI Appendix*, Tables S2–S6).

In the aεFab81/IgE-Fc, aεFab92/IgE-Fc, and aεFab98/IgE-Fc complexes, the interaction with the common epitope is formed largely by the V_H_ domain, and the binding sites share a number of features (Fig. 6*A* and *SI Appendix*, Figs. S4 *A*, *C*, and *E*). In the Lys302 binding pocket, Lys302 packs against Trp33 (CDRH1) and Tyr100B (CDRH3) (Chothia numbering) on one side, while the opposite side of the pocket is bordered by CDRH3 (Fig. 6*A*). With the exception of a Gly/Ala substitution at position 100, the CDRH3 sequence is identical in aεFab81 and aεFab92 (Fig. 1) and its conformation is similar in both complexes. In the aεFab81/IgE-Fc and aεFab92/IgE-Fc complexes, Asp95 (CDRH3) at the base of the Lys302 binding pocket forms a hydrogen bond with Lys302 (Fig. 6*A*). In the aεFab98/IgE-Fc complex, Asp95 is conservatively substituted for glutamic acid, which forms a salt bridge with Lys302.

The interfaces between the common epitope and the aεFab81, aεFab92, and aεFab98 V_L_ domains also share similar features (Fig. 6*B* and *SI Appendix*, Figs. S4 *B*, *D*, and *F*). For example, in all three complexes, His246 and Phe247 (Cε2) pack against CDRL3, with a hydrogen bond between Phe247 and Ser95 (CDRL3) (Fig. 6*B*).

The interface between aεFab67 and the common epitope is somewhat different and, in contrast to the other aεFabs, both V_H_ and V_L_ domains form the Lys302 binding pocket (Fig. 6*C* and *SI Appendix*, Fig. S5). However, key features of the binding pocket, such as the interaction with aromatic residues and an acidic residue at the base of the pocket that forms a hydrogen bond with Lys302, are conserved. Similarity in these key interactions is perhaps not surprising due to the high level of sequence similarity. In the aεFab67/IgE-Fc complex, one side of the Lys302 pocket is formed by Tyr33 (CDRH1) and Tyr50 (CDRH2/framework junction), with Asp95 (CDRH3) forming the base (Fig. 6*C*) (Chothia numbering). The other side of the pocket is bordered by CDRL3 residues (*SI Appendix*, Fig. S5).

In contrast to the other aεFabs, the interaction with the common epitope is formed largely by the aεFab67 V_L_ domain (Fig. 6*D* and *SI Appendix*, Fig. S5), and sequence differences alter the nature of the contacts. For example, Ser95 in aεFabs 81, 92, and 98 is substituted for Ile95 in aεFab67, with the loss of the hydrogen bond between the Ser95 side chain and Phe247 main chain (Fig. 6*D*). Furthermore, Glu95A (CDRL3) in aεFab67^A^ forms a salt bridge with His246 (Cε2) (Fig. 6*D*) (Glu95A in aεFab67^B^ forms a hydrogen bond with Arg427 from the Cε3 domain due to the asymmetric conformation adopted by IgE-Fc in this complex).

### IgE-Fc Adopts a Variety of Different Conformations When in Complex with the aεFabs.

**Fig. 7.**
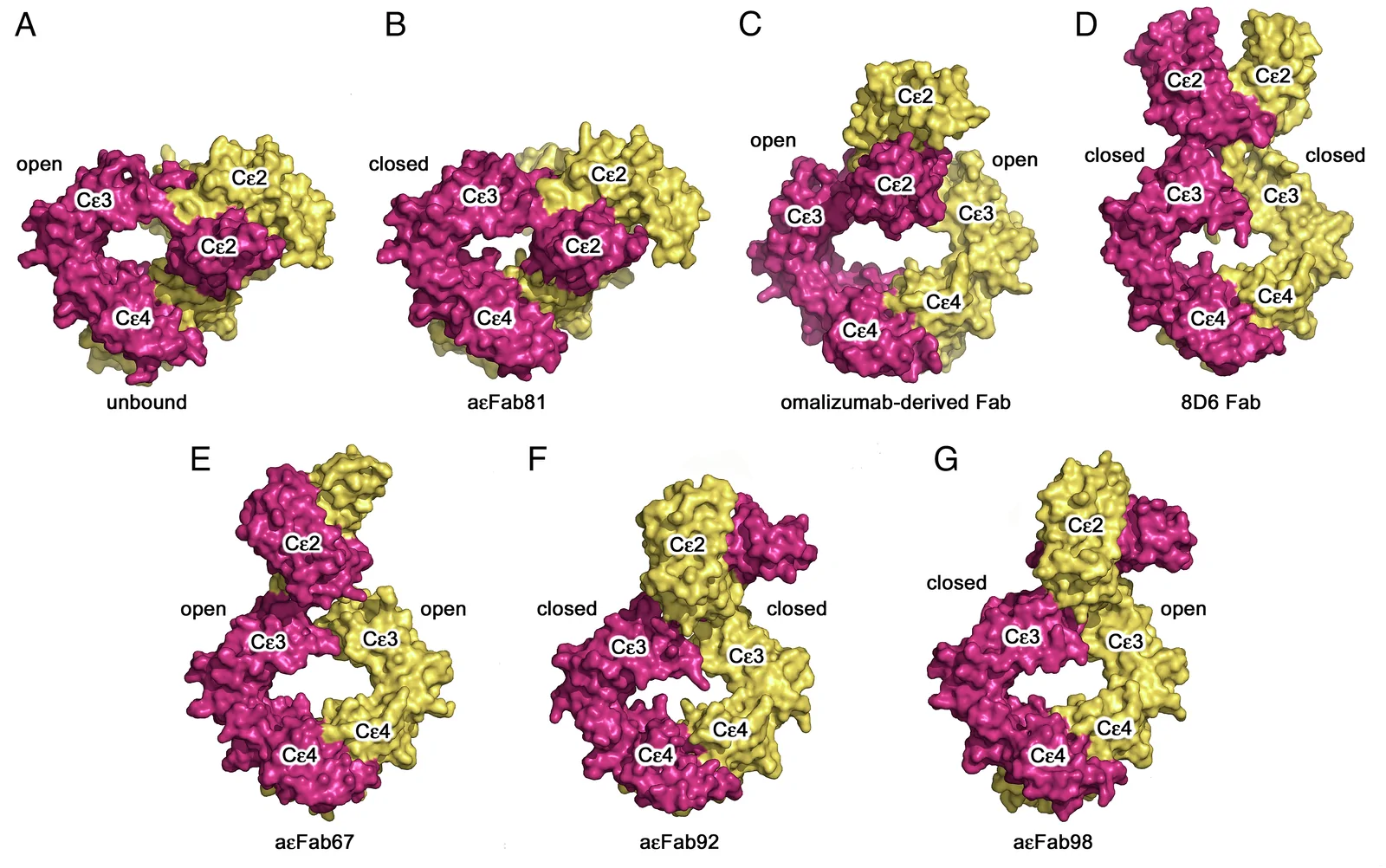
IgE-Fc conformations. (*A*) Unbound IgE-Fc adopts an acutely bent conformation (9, 10, 14). (*B*) Acutely bent conformation in the aεFab81/IgE-Fc complex. (*C*) Partially bent IgE-Fc conformation in the omalizumab-derived Fab/IgE-Fc complex (27). (*D*) Fully extended IgE-Fc conformation in the 8D6 Fab/IgE-Fc complex (8). (*E*) Partially extended conformation in the aεFab67/IgE-Fc complex. (*F*) Partially extended conformation in the aεFab92/IgE-Fc complex. (*G*) Partially extended conformation in the aεFab98/IgE-Fc complex. In panels *A*–*G*, IgE-Fc chains A and B are colored yellow and pink, respectively, and the “open” or “closed” conformation of the Cε3 domains in the aεFab complexes is indicated.

Although the aεFabs bind a common epitope on the Cε2 domain, IgE-Fc adopts a variety of different conformations when in complex with these Fabs (Figs. 4 and 7).

In the 2:1 aεFab67/IgE-Fc complex (Figs. 4 *A* and *B*, and 7*E*), IgE-Fc adopts an asymmetric, partially extended conformation, with a bend angle of ~151°, and the Cε3 domains adopt an open conformation. Due to the asymmetric conformation of IgE-Fc in the 2:1 complex with aεFab67, the interface with each Fab differs. Apart from the common epitope, aεFab67^A^ forms only a minor contact with the Cε3 domain in chain B. By contrast, aεFab67^B^ forms more extensive contacts with the Cε3 domain in each chain (*SI Appendix*, Fig. S6 *A* and *B*).

In contrast, IgE-Fc adopts an acutely bent conformation in the 1:1 aεFab81/IgE-Fc complex (Fig. 4 *C* and *D*), which is less acutely bent (~67°) than that for unbound IgE-Fc (62°) (9, 10, 14) and sFcεRIα-bound IgE-Fc (54°) (14), but more acutely bent than that in the 1:1 complex between IgE-Fc and sCD23 (76°) (15). In unbound IgE-Fc, the Cε3 domain in chains A and B adopt closed and open conformations, respectively (Fig. 7*A*). However, the opposite is observed in the aεFab81/IgE-Fc complex: the Cε3 domain in chain A is more open while that in chain B is closed (Fig. 7*B*).

Outside the common epitope, the aεFab81 V_H_ domain contacts the Cε2-proximal region of each Cε3 domain of IgE-Fc and the V_L_ domain contacts the Cε3 domain in chain B (*SI Appendix*, Fig. S6 *C* and *D*). The V_H_ domain also contacts the Cε2-Cε3 domain linker in chain A, where Glu55 (CDRH2) forms a salt bridge with Arg334.

IgE-Fc also adopts partially extended conformations in the 1:1 aεFab92/IgE-Fc and aεFab98/IgE-Fc complexes (Fig. 4 *E*–*H* and *SI Appendix,* Fig. S7*A*). Although the bend angle for IgE-Fc in these complexes is only ~20° smaller than that in the aεFab67/IgE-Fc complex (~129° and ~132° in the aεFab92/IgE-Fc and aεFab98/IgE-Fc complexes, respectively), they reveal large-scale conformational differences in the overall structure of IgE-Fc: in the aεFab92/IgE-Fc and aεFab98/IgE-Fc complexes, the (Cε2)_2_ domain pair undergoes a substantial rotation (Fig. 7 *F* and *G*), which is discussed in more detail below. Many of the contacts with IgE-Fc outside the common epitope are similar for both Fabs (Fig. 4 *E*–*H* and *SI Appendix*, Fig. S8) and their V_H_ domains contact the Cε2-Cε3 domain linker in chain A and the Cε3 domain in chain B.

A notable difference between the two complexes is the conformation adopted by the Cε3 domain in chain A: it is markedly open in the aεFab98/IgE-Fc complex and closed in the aεFab92/IgE-Fc complex (*SI Appendix*, Fig. S7 *B* and *C*). The transformation which closes the Cε3 domain in chain A in the aεFab92/IgE-Fc complex also rocks the (Cε2)_2_ domain pair, as a rigid unit, toward the Cε3 domain in chain B (Movie S1).

Even though these Fabs bind the same epitope, they adopt a variety of orientations relative to the Cε2 domain (*SI Appendix*, Fig. S9). aεFab81, 92, and 98 adopt a similar orientation but aεFab67 is markedly different. Intriguingly, the positions of the two aεFab67 molecules are not identical, suggesting that there is also some interplay between the asymmetric IgE-Fc conformation and the overall position adopted by each Fab.

### The (Cε2)_2_ Domain Pair Displays Substantial Rotational Flexibility.

In acutely bent IgE-Fc, the overall position of the (Cε2)_2_ domain pair is such that the Cε2 domain in chain B contacts the Cε3 domain in chain A (Fig. 7*A*). The (Cε2)_2_ domain pair is positioned between the Cε3 domains when IgE-Fc is partially (90°) bent (Fig. 7*C*) (27), and in the fully extended conformation, each Cε2 domain is closest to, or indeed contacts, only the Cε3 domain in the same chain (8, 21) (Fig. 7*D*).

In the aεFab67/IgE-Fc complex, IgE-Fc adopts a conformation along an unbending trajectory that lies between the partially bent conformation (Fig. 7 *A* and *B*) and the fully extended conformation (Fig. 7*D*). The transformation that generates the partially extended conformation in the aεFab67/IgE-Fc complex (Fig. 7*E*), involves further unbending of the (Cε2)_2_ domain pair and a clockwise rotation about its local two-fold axis (Movie S2), as the molecule is oriented with the (Cε2)_2_ domain pair facing toward the viewer. This transformation brings the Cε2 and Cε3 domains in each chain closer together.

A striking feature of the aεFab92/IgE-Fc and aεFab98/IgE-Fc complexes, in which IgE-Fc also adopts a partially extended conformation (Fig. 7 *F* and *G*), is that the (Cε2)_2_ domain pair undergoes a substantial anticlockwise rotation relative to the Cε3 and Cε4 domains as IgE-Fc unbends (Movie S3). This anticlockwise rotation, which we refer to as “twisting,” brings the Cε2 and Cε3 domains in different chains closer together.

### Structural Consequences of Single Amino Acid Residue Differences Between Fab Sequences.

Despite their high overall sequence similarity and recognition of a common epitope, comparison of the aεFab sequences reveal a small number of key amino acid differences that are consistent with the different functional activities of these Fabs. A notable difference between the Fabs is a glutamic acid/glycine substitution in CDRH2 (Fig. 1). In the aεFab81/IgE-Fc complex, Glu55 (CDRH2, Chothia numbering) forms a salt bridge with Arg334 in the Cε2–Cε3 linker and IgE-Fc adopts an acutely bent conformation (*SI Appendix,* Fig. S10*A*). In the other Fabs, Glu55 is substituted for glycine (*SI Appendix,* Fig. S10 *B–D*), which precludes the formation of an equivalent salt bridge with Arg334, and IgE-Fc instead adopts a variety of partially extended conformations.

Although the bend angle in IgE-Fc and the twist of the (Cε2)_2_ domain pair is similar in the aεFab98/IgE-Fc and aεFab92/IgE-Fc complexes (Figs. 4 *E*–*H* and 7 *E* and *F*), a striking functional difference is the ability of two aεFab92 molecules, but only a single aεFab98 molecule, to bind IgE-Fc (Fig. 2 *G*, *H*, *K*, and *L*). The aεFab92/IgE-Fc crystal structure does not reveal the full extent of the conformational flexibility that is possible in IgE-Fc when bound by this Fab as the binding site for a second aεFab92 molecule is obstructed. However, further unbending and rotation of the (Cε2)_2_ domain pair would render the site accessible. In these complexes, CDRH2 contains tyrosine (aεFab92)/valine (aεFab98) and glycine (aεFab92)/serine (aεFab98) substitutions (Fig. 1), and the position of the loop is shifted. The conformation of CDRH1, which contains an arginine (aεFab92)/serine (aεFab98) substitution, is in turn affected (*SI Appendix,* Fig. S11). CDRH2 contacts the Cε2–Cε3 linker and, depending on the conformation of IgE-Fc, has the ability to contact the Cε3 domain. Together with CDRH1, the position of CDRH2 could modulate the conformation of IgE-Fc as it flexes in solution, revealing or obscuring the binding site for a second Fab molecule.

## Discussion

As part of our studies to discover inhibitors of the functional and receptor binding activities of IgE, in particular the binding of IgE-Fc to the high-affinity receptor, FcεRI, we generated an extensive panel of anti-IgE-Fc antibodies. We found that four of these had very similar sequences both in their CDR and framework regions; although each was derived from a unique, single B cell, they had clear sequence similarities. We expected that the Fab regions of these four antibodies (aεFab67, 81, 92, and 98) would recognize very similar epitopes on IgE-Fc, and this we confirmed, but we were surprised to find that not only did they display markedly different binding properties, engaging diverse conformations of IgE-Fc with different affinities and stoichiometries, but they also exhibited different functional activities with respect to their ability to bind FcεRI-bound IgE-Fc and induce dissociation. These four antibodies thus challenge the conventional wisdom that antibodies with similar sequence binding to the same epitope will have similar functional activities.

Reflecting their notable sequence similarity (~88% framework identity outside of the CDRs), all four clones were isolated from a single immunized animal. This shared individual background makes a common ancestral lineage plausible, but not definitive. However, in the absence of a full high-throughput B cell repertoire analysis, our dataset lacks the somatic intermediate sequences necessary to trace a definitive lineage. It therefore remains an open question whether these antibodies arose from a single parental clone via diverging somatic hypermutation, or if they represent an example of convergent evolution within a single immune system driving toward a highly optimized mode of core epitope recognition.

IgE-Fc is a homodimer of Cε2, Cε3, and Cε4 domains, but free IgE-Fc is asymmetrically bent (9, 10, 14, 39, 41) and the two chains are conformationally distinct (Fig. 7*A*). As a consequence of this asymmetry, the common epitope recognized by the four aεFabs, at the base of the Cε2 domain, is accessible in one chain but partially buried in the other. It is therefore surprising that two of the Fabs, aεFab92 and aεFab98, engage the epitope that is partially buried in free IgE-Fc, even though an equivalent epitope is fully accessible in the other chain. Binding of these Fabs (and the second aεFab67 molecule) implies that free IgE-Fc must first undergo flexing in solution, transiently exposing the less accessible epitope. Indeed, we know from earlier Fab binding studies that IgE-Fc is dynamic (8, 21, 27) (Fig. 7 *C* and *D*) and molecular dynamics simulations suggest that partially extended conformations are relatively stable (21).

Not only is there a range of stoichiometry from a fully formed 2:1 complex (aεFab67), through mixtures of 2:1 and 1:1 complexes (aεFab92 and aεFab98), to 1:1 complex only (aεFab81), but there is a wide range of IgE-Fc bend angles from most unbent in the 2:1 complex with aεFab67 (~151°), through partially bent in the 1:1 complexes of aεFab98 and aεFab92 (~132° and ~129°) to almost fully bent in the 1:1 complex of aεFab81 (~67°; cf. 62° in unbound IgE-Fc). Strikingly, a single amino acid difference between aεFab81 and the other aεFabs appears to capture the acutely bent conformation observed in this complex.

The effect of binding each of the anti-IgE-Fc antibodies to receptor-bound IgE-Fc (in fact a complex between IgE-Fc and sFcεRIα) was tested, and again, each antibody behaved differently. aεFab67 was found to bind tightly and form a trimolecular 1:1:1 aεFab67/IgE-Fc/sFcεRIα complex, and aεFab67 and sFcεRIα showed positive cooperativity in their binding to IgE-Fc (*SI Appendix,* Fig. S3). In contrast, the three other Fabs display negative cooperativity toward the IgE-Fc/sFcεRIα complex, and caused induced dissociation of IgE-Fc from the receptor, aεFab81 to the greatest extent (Fig. 3 *B*, *Inset*), aεFab98 less so (Fig. 3 *D*, *Inset*), and aεFab92 almost not at all (Fig. 3 *C*, *Inset*). FcεRI binding requires both Cε3 domains to be in an open conformation, and this is the case in the aεFab67 complex (Fig. 7*E*). However, in the complex with aεFab98, the Cε3 domain in chain A is open and that in chain B is closed (Fig. 7*G*), while in the aεFab92 complex, both are closed (Fig. 7*F*).

Large-scale conformational flexibility is suggested to play an important role in the different biological functions of IgE, in which extended conformations of IgE-Fc could facilitate antigen capture by the IgE B cell receptor or influence antigen-mediated crosslinking of IgE bound to FcεRI on effector cells (21). Clockwise and anticlockwise (twisting) rotations of the (Cε2)_2_ domain pair would further expand the space available for the Fabs to explore. Indeed, in the structure of the human IgM B cell receptor (42, 43), IgM-Fc adopts a partially extended conformation that projects the Fabs away from the membrane, and the (Cμ2)_2_ domain pair is also twisted relative to the Cμ3 and Cμ4 domains. For these isotypes that lack a hinge region, a range of partially extended conformations and domain pair twisting in the Fc region could allow the B cell receptor to adopt the necessary architecture for antigen crosslinking.

Evidence presented here underscores that, for highly conformationally plastic molecules such as IgE, antibodies recognizing closely related epitopes can nevertheless elicit distinct functional outcomes. Many antibody discovery pipelines routinely employ early epitope binning to enhance epitope diversity, frequently excluding antibodies that bind to overlapping antigenic regions. However, because conformational flexibility and intrinsic disorder govern the distribution and coupling of conformational ensembles that mediate molecular interactions, prioritizing epitope diversity may paradoxically limit the functional diversity of antibody candidates. These limitations will be most pronounced when targeting dynamic proteins in which allosteric regulation arises from shifts within an interconnected energetic network rather than from fixed structural elements. Taken together, these observations highlight the importance of incorporating both structural flexibility and functional outcomes into antibody selection strategies, particularly when targeting dynamic proteins.

## Materials and Methods

### Anti-IgE Isolation and Protein Production.

Monoclonal anti-human IgE-Fc antibodies (αεFab67, αεFab81, αεFab92, and αεFab98) were isolated from immunized outbred Half-Lop rabbits under UK project license 30/2981 using single B cell screening and variable domain recovery. Recombinant Fabs were expressed in the Expi293 system and purified using sequential Protein G affinity and size-exclusion chromatography.

### SPR and Solution Complexes.

Kinetic binding profiles and sandwich assays were evaluated using a Biacore T200 instrument at 25 °C. All final surface plasmon resonance binding characteristics were validated across a minimum of three independent experimental replicates. Solution complexes were prepared at 1:1 and 2:1 (αεFab to IgE-Fc) molar ratios and evaluated via analytical size-exclusion chromatography using a Superdex 200 Increase column.

### Crystallization and Structure Determination.

Crystals of αεFab/IgE-Fc complexes were grown at 18 °C via sitting-drop vapor diffusion and cryoprotected prior to data collection. X-ray diffraction data were collected at Diamond Light Source beamlines I02, I03, and I04-1 and processed with XDS and the CCP4 suite (44, 45). Structures were solved by molecular replacement using coordinates from published IgE-Fc and Fab structures as search models (14, 21, 46). Atomic models were iteratively improved using interactive model building and automated structural refinement (47, 48).

### Detailed Protocols.

Complete step-by-step methodologies, specific buffer formulations, crystallization conditions, software programs, structural template entries, data processing, refinement and validation statistics, and numbering conversion tables are detailed comprehensively in *SI Appendix*, *Materials and Methods* and *SI Appendix*, Tables S1–S9.

## Supplementary Material

Appendix 01 (PDF)

Movie S1. Conformation of IgE-Fc in the aεFab98/IgE-Fc and aεFab92/IgE-Fc complexes. The movie shows a surface representation of IgE-Fc. IgE-Fc chains A and B are colored yellow and pink respectively. The movie begins by showing the partially extended structure for IgE-Fc in the aεFab98/IgE-Fc complex. This partially extended conformation is then morphed to the partially extended conformation in the aεFab92/IgE-Fc complex. Transformation between the two conformations involves closure of the Cε3 domain in chain A and rocking of the (Cε2)_2_ domain pair towards the Cε3 domain in chain B.

Movie S2. Rotation of the (Cε2)_2_ domain pair in the aεFab67/IgE-Fc complex. The movie shows a surface representation of IgE-Fc. IgE-Fc chains A and B are colored yellow and pink respectively. The movie begins by showing the acutely bent structure for unbound IgE-Fc. The acutely bent conformation is morphed to the partially bent conformation observed in the omalizumab-derived Fab/IgE-Fc complex, and then to the partially extended conformation in the aεFab67/IgE-Fc complex. Transformation from the partially bent to the partially extended conformation involves closure of both Cε3 domains and rocking of the (Cε2)_2_ domain pair towards the Cε3 domain in chain B.

Movie S3. Rotation of the (Cε2)_2_ domain pair in the aεFab98/IgE-Fc complex. The movie shows a surface representation of IgE-Fc. IgE-Fc chains A and B are colored yellow and pink respectively. The movie begins by showing the acutely bent structure for unbound IgE-Fc. The acutely bent conformation is morphed to the partially bent conformation observed in the omalizumab-derived Fab/IgE-Fc complex, and then to the partially extended conformation observed in the aεFab98/IgE-Fc complex. Transformation from the partially bent to the partially extended conformation involves closure of the Cε3 domain in chain B and an anti-clockwise rotation of the (Cε2)_2_ domain pair.

## Data Availability

Coordinates and structure factors have been deposited at the Protein Data Bank with accession numbers pdb_000030AC (49), aεFab67/IgE-Fc complex; pdb_000030AD (50), aεFab81/IgE-Fc complex; pdb_000030AE (51), aεFab92/IgE-Fc complex; and pdb_000030AF (52), aεFab98/IgE-Fc complex.
