# Supplementary material for: Antibodies targeting a shared epitope exploit IgE allostery to drive distinct functional outcomes
Source: Proc Natl Acad Sci U S A. 2026 Sep 8;123(37):e2613091123. doi: 10.1073/pnas.2613091123 (PMC13578773; doi:10.1073/pnas.2613091123)
Supplement: Supplementary file 1 [file pnas.2613091123.sapp.pdf]

# Supporting Information for

## Antibodies targeting a shared epitope exploit IgE allostery to drive distinct functional outcomes

Anna M. Davies[a], Nyssa Drinkwater[a], Andrew J. Beavil[a], Victoria O'Dowd[b], Daniel Lightwood[b], Tom Ceska[b], Alistair J. Henry[b], Brian J. Sutton[a] and James M. McDonnell[a]*

[a]Randall Centre for Cell and Molecular Biophysics, King's College London, New Hunt's House, Guy's Campus, London, SE1 1UL, UK; [b]UCB, Slough, UK.

*Corresponding author: James M. McDonnell.

**Email:** james.mcdonnell@kcl.ac.uk.

**This PDF file includes:**

Materials and Methods
Tables S1 to S9
Figures S1 to S11
Legends for Movies S1 to S3
SI References

**Other supplementary materials for this manuscript include the following:**

Movies S1 to S3

## Materials and Methods

**Anti-IgE Discovery.** Four outbred female Half-Lop rabbits were immunized subcutaneously with up to five doses of 500 μg human IgE-Fc protein per injection. The IgE-Fc was emulsified in a 1:1 volume of Complete Freund's adjuvant (CFA). All animal procedures were performed at UCB (Slough, UK) in accordance with the UK Animals (Scientific Procedures) Act 1986 and were approved by the local Animal Welfare and Ethical Review Body (AWERB), conducted under project licence 30/2981. The four distinct anti-IgE antibody clones described in this study (aεFab67, aεFab81, aεFab92 and aεFab98) were all subsequently isolated from a single individual animal from this cohort. Antigen-specific monoclonal antibodies were generated according to previously described protocols (1,2). Splenocytes and peripheral blood mononuclear cells (PBMCs) were isolated and cultured for 7 days. Supernatants were screened for binding to IgE-Fc using a fluorescence microvolume assay technology (FMAT) on an Applied Biosystems 8200 Cellular Detection System (ABI). Positive hits from master plates were further validated for target binding and FcεRI receptor blocking via enzyme-linked immunosorbent assay (ELISA) and surface plasmon resonance (SPR). Antigen-specific single B-cells were isolated from positive wells using the fluorescent foci method (1). Heavy and light chain variable region genes ($V_H$ and $V_L$) were recovered via RT-PCR, cloned and sequenced. Finally, paired recombinant antibodies were expressed and purified for further testing.

**Cloning, protein expression and purification.** IgE-Fc was produced as described previously (3). Anti-IgE Fabs were produced using the Expi293$^{TM}$ expression system (Gibco, Life Technologies). Transfections were performed according to the manufacturer's instructions. Fabs were initially purified on a HiTrap Protein G HP column (Cytiva), and then by size exclusion chromatography (SEC) on a Superdex 26/60 S200 column (Cytiva) in PBS pH 7.4.

**Surface Plasmon Resonance.** Experiments were performed on a Biacore T200 instrument (Cytiva) in 10 mM HEPES (pH 7.4), 150 mM NaCl, 0.005% (v/v) surfactant P20 as running buffer at flow rates of 30 µL/min at 25 °C. In direct binding experiments, Fabs (aεFab67, aεFab81, aεFab92 and aεFab98) were immobilized on a CM5 sensor chip (Cytiva) using standard amine coupling protocols, where coupling densities were kept relatively low (<300 resonance units). This was followed by binding experiments of IgE-Fc to the immobilized Fabs, using two-fold dilution series of IgE-Fc, each performed in duplicate; typically, the association phases were monitored for 240-300 s, followed by 900 s of dissociation. The surfaces were regenerated by two 60 s pulses of 100 mM glycine-HCl, pH 2.2. For sandwich binding experiments, the same Fab surfaces described above were utilized, but ~100 resonance units of IgE-Fc were first captured, followed by the binding of a two-fold dilution series of Fabs (aεFab67, aεFab81, aεFab92 and aεFab98), to assess the binding characteristics of the second Fab of a 2:1 (Fab/IgE-Fc) complex. Standard double referencing experiments (4) were utilized to generate binding curves, and kinetic fits were performed using Origin software (OriginLab). To ensure robustness, assay conditions were optimized across multiple independent exploratory runs, and all final SPR binding characteristics were confirmed across a minimum of three independent experimental replicates. Representative datasets executed with technical duplicates are displayed. The error values reported in Table S1 represent the standard errors derived from the global kinetic fitting of these optimized datasets, with example kinetic overlays shown in Figure S2.

**Size exclusion chromatography of aεFab/IgE-Fc complexes.** IgE-Fc was mixed with aεFab67, aεFab81, aεFab92 and aεFab98 with aεFab/IgE-Fc molar ratios of 1:1 and 2:1. The aεFab/IgE-Fc complexes, in PBS pH 7.4 and 0.1% (w/v) sodium azide, were incubated at room temperature for 2-3 hours before loading onto a Superdex 200 Increase 10/300 GL column (Cytiva) that had been equilibrated with PBS pH 7.4 and 0.1% (w/v) sodium azide.

**Crystallization.** All crystals were grown at 18 °C using the sitting drop vapor diffusion method. For the aεFab67/IgE-Fc complex, the proteins were mixed in a 2:1 ratio and diluted to a concentration

of 3.5 mg/mL with a buffer comprising 20 mM Tris-HCl pH 7.5 and 25 mM NaCl. Crystals were grown in 0.1 M Tris-HCl pH 7.5 and 18% (w/v) PEG 8000, using a reservoir volume of 70 µL and drops comprising 1 µL protein solution and 1 µL reservoir. Crystals were cryoprotected in 0.1 M Tris-HCl pH 8, 20% (w/v) PEG 8000 and 30% (v/v) ethylene glycol. For the aεFab81/IgE-Fc complex, the proteins were mixed in a 1:1 ratio and diluted to a concentration of 5.2 mg/mL with a buffer comprising 20 mM Tris-HCl pH 7.5 and 25 mM NaCl. Crystals were grown in 0.1 M Tris-HCl pH 8.5 and 30% (v/v) PEG 400, using a reservoir volume of 70 µL and drops comprising 500 nL protein solution and 250 nL reservoir. Crystals were cryoprotected using the mother liquor. For the aεFab92/ IgE-Fc complex, the proteins were mixed in a 1:1 ratio and diluted to a concentration of 3 mg/mL with a buffer comprising 20 mM Tris-HCl pH 7.5 and 25 mM NaCl. Crystals were grown in 0.1 M sodium citrate pH 5.5, 12% (w/v) PEG 6000, 0.1 M ammonium chloride and 4% (v/v) ethylene glycol, using a reservoir volume of 70 µL and drops comprising 500 nL protein solution and 750 nL reservoir. Crystals were cryoprotected in 0.1 M sodium citrate pH 5.5, 12% (w/v) PEG 6000, 0.1 M ammonium chloride and 30% (v/v) ethylene glycol. For the aεFab98/IgE-Fc complex, the proteins were mixed in a 1:1 ratio and diluted to a concentration of 3.1 mg/mL with a buffer comprising 20 mM Tris-HCl pH 7.5 and 20 mM NaCl. Crystals were grown in 0.1 M sodium cacodylate pH 6.5, 8% (w/v) PEG 8000 and 0.1 M calcium acetate, using a reservoir volume of 70 µL and drops comprising 1000 nL protein solution and 500 nL reservoir. Crystals were cryoprotected in 0.1 M sodium cacodylate pH 6.5, 8% (w/v) PEG 8000, 0.1 M calcium acetate and 25% (v/v) glycerol.

**X-ray diffraction data collection and processing.** X-ray diffraction data were collected at the Diamond Light Source (Harwell, UK), at beamlines I02 (aεFab67 and aεFab92), I03 (aεFab81) and I04-1 (aεFab98). Data were integrated with XDS (5) using the xia2 package (6) and scaled with AIMLESS from the CCP4 suite (7,8).

**Structure determination, model building and refinement.** Structures were solved by molecular replacement with PHASER (9) using protein atoms from PDB entries pdb_00002wqr (10) and pdb_00004j4p (11) as search models. Refinement was performed with PHENIX (12) and alternated with interactive model building in Coot (13). The quality of the models was assessed with MolProbity (14). Data processing and refinement statistics are presented in *SI Appendix*, Table S7. Interfaces were analyzed with PISA (15), and figures and movies were prepared with PyMOL (16) and Chimera (17).

aεFab variable domain residues were numbered according to the Chothia numbering system using the AbNum server (18) and this numbering system is used throughout the text. However, aεFab residues are numbered sequentially in the Protein Data Bank entries for each aεFab/IgE-Fc complex. Both numbering schemes are presented in SI Appendix, Tables S8 and S9.

**Table S1.** Binding kinetics and affinities for four anti-IgE-Fc Fabs

| | $K_D$ (pM) | $k_{on}$ ($\times 10^5\ M^{-1}\ s^{-1}$) | $k_{off}$ ($\times 10^{-5}\ s^{-1}$) | $K_{D2}$ (pM) | $k_{on2}$ ($\times 10^5\ M^{-1}\ s^{-1}$) | $k_{off2}$ ($\times 10^{-5}\ s^{-1}$) |
|---|---|---|---|---|---|---|
| **aεFab67** | 300 | 9.8 (± 0.1) | 30 (± 0.8) | 4600 | 1.2 (± 0.1) | 550 (± 12) |
| **aεFab81** | 15 | 1.3 (± 0.1) | 1.8 (± 0.1) | | | |
| **aεFab92** | 40 | 7.4 (± 0.1) | 3.0 (± 0.1) | | | |
| **aεFab98** | 380 | 9.0 (± 0.1) | 33 (± 0.5) | 3400 | 1.9 (± 0.1) | 660 (± 16) |

**Table S2.** List of interfacing residues in the aεFab67/IgE-Fc complex: aεFab67[A] and IgE-Fc.

| aεFab67[A] | | IgE-Fc | |
|---|---|---|---|
| **Chain** | **Residue** | **Chain** | **Residue** |
| C | Thr31 | A | His246 |
| C | Tyr33 | A | Phe247 |
| C | Trp47 | A | Pro249 |
| C | Tyr50 | A | Thr250 |
| C | Tyr52 | A | Asp271 |
| C | Ser54 | A | Met275 |
| C | Ser56 | A | Asp276 |
| C | Tyr58 | A | Asp278 |
| C | Asn61 | A | Leu279 |
| C | Trp62 | A | Thr298 |
| C | Lys64 | A | Leu299 |
| C | Gly65 | A | Ser300 |
| C | Arg66 | A | Gln301 |
| C | Asp95 | A | Lys302 |
| C | Pro96 | A | His303 |
| C | Tyr98 | A | Leu305 |
| C | Ser99 | A | Ser306 |
| D | Ala1 | A | Asp307 |
| D | Leu2 | A | Arg308 |
| D | Gln27 | A | Lys327 |
| D | Ser28 | B | Ala329 |
| D | Ile29 | B | Asp330 |
| D | Gly30 | B | Pro423 |
| D | Tyr32 | B | His424 |
| D | Lys50 | B | Pro426 |
| D | Gln89 | B | Arg427 |
| D | Asp91 | | |
| D | Thr92 | | |
| D | Ala93 | | |
| D | Val94 | | |
| D | Ile95 | | |
| D | Glu95A | | |
| D | Glu95C | | |

**Table S3.** List of interfacing residues in the aεFab67/IgE-Fc complex: aεFab67[B] and IgE-Fc.

| **aεFab67[B]** | | **IgE-Fc** | |
|---|---|---|---|
| **Chain** | **Residue** | **Chain** | **Residue** |
| H | Arg10 | A | Ser331 |
| H | Thr13 | A | Ala377 |
| H | Pro14 | A | Gly413 |
| H | Gly15 | A | Thr415 |
| H | Thr16 | A | Gln417 |
| H | Pro17 | A | Arg427 |
| H | Thr31 | A | Ala428 |
| H | Tyr33 | A | Leu429 |
| H | Tyr50 | A | Met430 |
| H | Tyr52 | A | Arg431 |
| H | Gly53 | A | Ser432 |
| H | Ser54 | A | Thr433 |
| H | Gly55 | A | Thr434 |
| H | Ser56 | A | Ser437 |
| H | Tyr58 | B | His246 |
| H | Asn61 | B | Phe247 |
| H | Lys64 | B | Pro249 |
| H | Gly65 | B | Thr250 |
| H | Arg66 | B | Asp276 |
| H | Thr68 | B | Asp278 |
| H | Ser70 | B | Leu279 |
| H | Lys71 | B | Thr298 |
| H | Thr81 | B | Leu299 |
| H | Ser82 | B | Ser300 |
| H | Asp96 | B | Gln301 |
| H | Tyr99 | B | Lys302 |
| L | Leu2 | B | His303 |
| L | Gln27 | B | Leu305 |
| L | Ser28 | B | Ser306 |
| L | Ile29 | B | Asp307 |
| L | Gly30 | B | Arg308 |
| L | Ser31 | B | Pro365 |
| L | Tyr32 | B | Lys391 |
| L | Lys50 | B | Gln392 |

| | | | |
|---|---|---|---|
| L | Gln89 | B | Arg393 |
| L | Gln90 | B | Asn394 |
| L | Asp91 | B | Gly395 |
| L | Thr92 | | |
| L | Ala93 | | |
| L | Val94 | | |
| L | Ile95 | | |
| L | Glu95A | | |
| L | Thr97 | | |

**Table S4.** List of interfacing residues in the aεFab81/IgE-Fc complex.

| aεFab81 | | IgE-Fc | |
|---|---|---|---|
| **Chain** | **Residue** | **Chain** | **Residue** |
| H | Glu15 | A | Gly245 |
| H | Ser31 | A | His246 |
| H | Thr31A | A | Phe247 |
| H | Trp33 | A | Pro249 |
| H | Val52 | A | Thr250 |
| H | Gly53 | A | Ser300 |
| H | Ser54 | A | Gln301 |
| H | Glu55 | A | Lys302 |
| H | Asn56 | A | His303 |
| H | Thr57 | A | Leu305 |
| H | Tyr58 | A | Ser306 |
| H | Tyr59 | A | Asp307 |
| H | Ser61 | A | Arg308 |
| H | Trp62 | A | Lys327 |
| H | Glu64 | A | Asp330 |
| H | Gly65 | A | Arg334 |
| H | Arg66 | A | Pro426 |
| H | Ser70 | A | Arg427 |
| H | Lys71 | B | Ala329 |
| H | Ser82A | B | Asp330 |
| H | Leu82B | B | Ser331 |
| H | Thr83 | B | Pro333 |
| H | Asp95 | B | Arg334 |
| H | Ser97 | B | Ala364 |
| H | Asp98 | B | Pro365 |
| H | Ser99 | B | Ser366 |
| H | Gly100 | B | Lys367 |
| H | Asp100A | B | Gly368 |
| H | Tyr100B | B | Pro423 |
| L | Ala1 | B | His424 |
| L | Tyr2 | B | Pro426 |
| L | Asp3 | B | Arg427 |
| L | Ser28 | | |

| | |
|---|---|
| L | Tyr32 |
| L | Gly91 |
| L | Ile92 |
| L | Thr93 |
| L | Ile94 |
| L | Ser95 |
| L | Gly95A |
| L | Glu95C |

**Table S5.** List of interfacing residues in the aεFab92/IgE-Fc complex.

| aεFab92 | | IgE-Fc | |
|---|---|---|---|
| **Chain** | **Residue** | **Chain** | **Residue** |
| H | Arg31 | A | Ser331 |
| H | Ser31A | A | Asn332 |
| H | Trp33 | A | Pro333 |
| H | Ile51 | A | Ser366 |
| H | Tyr52 | A | Lys367 |
| H | Ile52A | A | Gly368 |
| H | Gly53 | A | Pro423 |
| H | Ser54 | A | His424 |
| H | Gly55 | A | Pro426 |
| H | Ser56 | B | Gly245 |
| H | Thr57 | B | His246 |
| H | Tyr58 | B | Phe247 |
| H | Lys64 | B | Pro248 |
| H | Gly65 | B | Pro249 |
| H | Ser70 | B | Thr250 |
| H | Lys71 | B | Gln252 |
| H | Thr72 | B | Ser300 |
| H | Ser73 | B | Gln301 |
| H | Asp98 | B | Lys302 |
| H | Ser99 | B | His303 |
| H | Ala100 | B | Leu305 |
| H | Asp100A | B | Ser306 |
| H | Tyr100B | B | Asp307 |
| L | Tyr2 | B | Arg308 |
| L | Asn28 | B | Lys327 |
| L | Tyr30 | B | Pro426 |
| L | Asn32 | B | Arg427 |
| L | Gly91 | | |
| L | Ala92 | | |
| L | Thr93 | | |
| L | Ile94 | | |
| L | Ser95 | | |
| L | Gly95A | | |

**Table S6.** List of interfacing residues in the aεFab98/IgE-Fc complex.

| aεFab98 | | IgE-Fc | |
|---|---|---|---|
| **Chain** | **Residue** | **Chain** | **Residue** |
| H | Ser30 | A | Ala329 |
| H | Ser31A | A | Asp330 |
| H | Trp33 | A | Ser331 |
| H | Val52 | A | Asn332 |
| H | Ser53 | A | Pro333 |
| H | Ser54 | A | Arg334 |
| H | Gly55 | A | Ala364 |
| H | Ser56 | A | Pro365 |
| H | Thr57 | A | Ser366 |
| H | Tyr58 | A | Arg393 |
| H | Ser61 | A | Asn394 |
| H | Lys64 | A | Gly395 |
| H | Gly65 | A | Thr396 |
| H | Arg66 | B | Gly245 |
| H | Lys71 | B | His246 |
| H | Thr72 | B | Phe247 |
| H | Ser73 | B | Pro249 |
| H | Ser74 | B | Thr250 |
| H | Thr82 | B | Ser300 |
| H | Ser82A | B | Gln301 |
| H | Glu95 | B | Lys302 |
| H | Ser96 | B | His303 |
| H | Val97 | B | Leu305 |
| H | Gly98 | B | Ser306 |
| H | Ser99 | B | Asp307 |
| H | Ala100 | B | Arg308 |
| H | Tyr100A | B | Lys327 |
| H | Tyr100B | B | Pro426 |
| L | Tyr2 | B | Arg427 |
| L | Tyr92 | | |
| L | Ser93 | | |
| L | Arg94 | | |
| L | Ser95 | | |
| L | Gly95A | | |

**Table S7.** Data processing and refinement statistics.

| **Data processing** | | | | |
|---|---|---|---|---|
| | **aεFab67** | **aεFab81** | **aεFab92** | **aεFab98** |
| Space group | $P\ 2_1\ 2_1\ 2_1$ | $P\ 3_1\ 2\ 1$ | $C\ 1\ 2\ 1$ | $C\ 1\ 2\ 1$ |
| Unit cell dimensions (Å) | *a* = 83.33<br>*b* = 117.54<br>*c* = 191.98 | *a* = 165.79<br>*b* = 165.79<br>*c* = 184.53 | *a* = 169.53<br>*b* = 86.84<br>*c* = 108.79<br>β = 110.17 | *a* = 163.36<br>*b* = 92.99<br>*c* = 105.08<br>β = 108.87 |
| Resolution (Å)[a] | 100.25-3.20<br>(3.37-3.20)[b] | 143.58-3.20<br>(3.31-3.20) | 46.80-3.50<br>(3.83-3.50) | 79.68-2.40<br>(2.47-2.40) |
| Completeness (%)[a] | 100 (99.7)[b] | 100 (100) | 99.6 (98.5) | 98.8 (99.4) |
| Multiplicity[a] | 11.8 (11.9)[b] | 10.2 (10.5) | 3.4 (3.2) | 3.5 (3.5) |
| Mean ((I)/σ(I))[a] | 10.3 (2.1)[b] | 14.4 (2.0) | 8.2 (1.4) | 10.8 (1.3) |
| $CC_{1/2}$[a] | 0.993 (0.565)[b] | 0.995 (0.651) | 0.980 (0.902) | 0.995 (0.694) |
| $R_{merge}$ (%)[a] | 31.3 (255.2)[b] | 13.8 (164.5) | 8.8 (73.2) | 7.3 (89.8) |
| $R_{pim}$ (%)[a] | 9.6 (79.2)[b] | 4.6 (53.3) | 5.6 (48.2) | 4.6 (55.9) |
| **Refinement** | | | | |
| $R_{work}$ / $R_{free}$ (%)[c] | 20.36 / 25.81 | 18.20 / 21.98 | 24.27 / 28.97 | 19.43 / 22.13 |
| No. of reflections | 31 808 | 48 831 | 18 537 | 57 496 |
| RMSD | | | | |
| Bond lengths (Å) | 0.002 | 0.004 | 0.003 | 0.003 |
| Bond angles (°) | 0.493 | 0.632 | 0.543 | 0.656 |
| Coordinate error (Å) | 0.43 | 0.40 | 0.81 | 0.31 |
| No. of atoms | | | | |
| Protein | 11 005 | 8 132 | 7 654 | 7 946 |
| Oligosaccharide | 122 | 177 | 119 | 122 |
| Solvent | 0 | 0 | 0 | 167 |
| Other | 26[d] | 49[e] | 0 | 86[f] |
| Ramachandran plot | | | | |
| Favoured (%) | 97.10 | 96.50 | 96.46 | 97.51 |
| Allowed (%) | 2.84 | 3.50 | 3.54 | 2.40 |

[a] Values in parentheses are for the highest resolution shell
[b] Three datasets collected from a single crystal were merged
[c] $R_{free}$ set comprises 5% of reflections
[d] Polyethylene glycol
[e] Polyethylene glycol, tris (hydroxymethyl) aminomethane
[f] Acetate, glycerol, polyethylene glycol

**Table S8.** Chothia and sequential numbering systems for the aεFab $V_H$ domains.

| aεFab67 | | | aεFab81 | | | aεFab92 | | | aεFab98 | | |
|---|---|---|---|---|---|---|---|---|---|---|---|
| **Residue** | **Chothia** | **Sequential** | **Residue** | **Chothia** | **Sequential** | **Residue** | **Chothia** | **Sequential** | **Residue** | **Chothia** | **Sequential** |
| Q | 2 | 1 | Q | 1 | 1 | Q | 2 | 1 | Q | 1 | 1 |
| S | 3 | 2 | E | 2 | 2 | S | 3 | 2 | E | 2 | 2 |
| L | 4 | 3 | Q | 3 | 3 | L | 4 | 3 | Q | 3 | 3 |
| E | 5 | 4 | L | 4 | 4 | E | 5 | 4 | L | 4 | 4 |
| E | 6 | 5 | E | 5 | 5 | E | 6 | 5 | E | 5 | 5 |
| S | 7 | 6 | E | 6 | 6 | S | 7 | 6 | E | 6 | 6 |
| G | 8 | 7 | S | 7 | 7 | G | 8 | 7 | S | 7 | 7 |
| G | 9 | 8 | G | 8 | 8 | G | 9 | 8 | G | 8 | 8 |
| R | 10 | 9 | G | 9 | 9 | D | 10 | 9 | G | 9 | 9 |
| L | 11 | 10 | D | 10 | 10 | L | 11 | 10 | D | 10 | 10 |
| V | 12 | 11 | L | 11 | 11 | V | 12 | 11 | L | 11 | 11 |
| T | 13 | 12 | V | 12 | 12 | K | 13 | 12 | V | 12 | 12 |
| P | 14 | 13 | K | 13 | 13 | P | 14 | 13 | K | 13 | 13 |
| G | 15 | 14 | P | 14 | 14 | E | 15 | 14 | P | 14 | 14 |
| T | 16 | 15 | E | 15 | 15 | G | 16 | 15 | E | 15 | 15 |
| P | 17 | 16 | G | 16 | 16 | S | 17 | 16 | G | 16 | 16 |
| L | 18 | 17 | S | 17 | 17 | L | 18 | 17 | S | 17 | 17 |
| T | 19 | 18 | L | 18 | 18 | T | 19 | 18 | L | 18 | 18 |
| L | 20 | 19 | T | 19 | 19 | L | 20 | 19 | T | 19 | 19 |
| T | 21 | 20 | L | 20 | 20 | T | 21 | 20 | L | 20 | 20 |
| C | 22 | 21 | T | 21 | 21 | C | 22 | 21 | T | 21 | 21 |
| T | 23 | 22 | C | 22 | 22 | T | 23 | 22 | C | 22 | 22 |
| A | 24 | 23 | T | 23 | 23 | A | 24 | 23 | T | 23 | 23 |
| S | 25 | 24 | A | 24 | 24 | S | 25 | 24 | A | 24 | 24 |
| G | 26 | 25 | S | 25 | 25 | G | 26 | 25 | S | 25 | 25 |
| F | 27 | 26 | G | 26 | 26 | I | 27 | 26 | G | 26 | 26 |
| S | 28 | 27 | F | 27 | 27 | S | 28 | 27 | F | 27 | 27 |
| L | 29 | 28 | S | 28 | 28 | F | 29 | 28 | S | 28 | 28 |
| S | 30 | 29 | V | 29 | 29 | S | 39 | 29 | F | 29 | 29 |
| T | 31 | 30 | S | 30 | 30 | R | 31 | 30 | S | 30 | 30 |
| Y | 32 | 31 | S | 31 | 31 | S | 31A | 31 | S | 31 | 31 |
| Y | 33 | 32 | T | 31A | 32 | Y | 32 | 32 | S | 31A | 32 |
| M | 34 | 33 | D | 32 | 33 | W | 33 | 33 | Y | 32 | 33 |
| S | 35 | 34 | W | 33 | 34 | I | 34 | 34 | W | 33 | 34 |
| W | 36 | 35 | I | 34 | 35 | W | 35 | 35 | I | 34 | 35 |
| V | 37 | 36 | W | 35 | 36 | W | 36 | 36 | C | 35 | 36 |
| R | 38 | 37 | W | 36 | 37 | V | 37 | 37 | W | 36 | 37 |
| Q | 39 | 38 | V | 37 | 38 | R | 38 | 38 | V | 37 | 38 |
| A | 40 | 39 | R | 38 | 39 | Q | 39 | 39 | R | 38 | 39 |
| P | 41 | 40 | Q | 39 | 40 | A | 40 | 40 | Q | 39 | 40 |
| G | 42 | 41 | A | 40 | 41 | P | 41 | 41 | A | 40 | 41 |
| K | 43 | 42 | P | 41 | 42 | G | 42 | 42 | P | 41 | 42 |
| G | 44 | 43 | G | 42 | 43 | K | 43 | 43 | G | 42 | 43 |
| L | 45 | 44 | K | 43 | 44 | G | 44 | 44 | K | 43 | 44 |
| E | 46 | 45 | G | 44 | 45 | L | 45 | 45 | G | 44 | 45 |
| W | 47 | 46 | L | 45 | 46 | E | 46 | 46 | L | 45 | 46 |
| I | 48 | 47 | E | 46 | 47 | W | 47 | 47 | E | 46 | 47 |
| G | 49 | 48 | W | 47 | 48 | I | 48 | 48 | W | 47 | 48 |
| Y | 50 | 49 | I | 48 | 49 | G | 49 | 49 | I | 48 | 49 |
| I | 51 | 50 | G | 49 | 50 | C | 50 | 50 | G | 49 | 50 |
| Y | 52 | 51 | C | 50 | 51 | I | 51 | 51 | C | 50 | 51 |
| T | 52A | 52 | I | 51 | 52 | Y | 52 | 52 | I | 51 | 52 |
| G | 53 | 53 | V | 52 | 53 | I | 52A | 53 | V | 52 | 53 |
| S | 54 | 54 | S | 52A | 54 | G | 53 | 54 | V | 52A | 54 |
| G | 55 | 55 | G | 53 | 55 | S | 54 | 55 | S | 53 | 55 |
| S | 56 | 56 | S | 54 | 56 | G | 55 | 56 | S | 54 | 56 |
| T | 57 | 57 | E | 55 | 57 | S | 56 | 57 | G | 55 | 57 |
| Y | 58 | 58 | N | 56 | 58 | T | 57 | 58 | S | 56 | 58 |
| Y | 59 | 59 | T | 57 | 59 | Y | 58 | 59 | T | 57 | 59 |
| A | 60 | 60 | Y | 58 | 60 | Y | 59 | 60 | Y | 58 | 60 |
| N | 61 | 61 | Y | 59 | 61 | A | 60 | 61 | Y | 59 | 61 |
| W | 62 | 62 | A | 60 | 62 | S | 61 | 62 | A | 60 | 62 |
| A | 63 | 63 | S | 61 | 63 | W | 62 | 63 | S | 61 | 63 |
| K | 64 | 64 | W | 62 | 64 | A | 63 | 64 | W | 62 | 64 |
| G | 65 | 65 | A | 63 | 65 | K | 64 | 65 | A | 63 | 65 |
| R | 66 | 66 | E | 64 | 66 | G | 65 | 66 | K | 64 | 66 |
| F | 67 | 67 | G | 65 | 67 | R | 66 | 67 | G | 65 | 67 |

| | | | | | | | | | | | |
|---|---|---|---|---|---|---|---|---|---|---|---|
| T | 68 | 68 | R | 66 | 68 | F | 67 | 68 | R | 66 | 68 |
| I | 69 | 69 | F | 67 | 69 | T | 68 | 69 | F | 67 | 69 |
| S | 70 | 70 | I | 68 | 70 | I | 69 | 70 | T | 68 | 70 |
| K | 71 | 71 | I | 69 | 71 | S | 70 | 71 | I | 69 | 71 |
| T | 72 | 72 | S | 70 | 72 | K | 71 | 72 | S | 70 | 72 |
| S | 73 | 73 | K | 71 | 73 | T | 72 | 73 | K | 71 | 73 |
| T | 74 | 74 | A | 72 | 74 | S | 73 | 74 | T | 72 | 74 |
| T | 75 | 75 | S | 73 | 75 | S | 74 | 75 | S | 73 | 75 |
| V | 76 | 76 | S | 74 | 76 | T | 75 | 76 | S | 74 | 76 |
| D | 77 | 77 | T | 75 | 77 | T | 76 | 77 | T | 75 | 77 |
| L | 78 | 78 | T | 76 | 78 | V | 77 | 78 | T | 76 | 78 |
| K | 79 | 79 | V | 77 | 79 | T | 78 | 79 | V | 77 | 79 |
| M | 80 | 80 | T | 78 | 80 | L | 79 | 80 | T | 78 | 80 |
| T | 81 | 81 | L | 79 | 81 | Q | 80 | 81 | L | 79 | 81 |
| S | 82 | 82 | Q | 80 | 82 | M | 81 | 82 | Q | 80 | 82 |
| P | 82A | 83 | M | 81 | 83 | T | 82 | 83 | M | 81 | 83 |
| T | 83 | 84 | T | 82 | 84 | S | 82A | 84 | T | 82 | 84 |
| T | 84 | 85 | S | 82A | 85 | L | 82B | 85 | S | 82A | 85 |
| E | 85 | 86 | L | 82B | 86 | T | 83 | 86 | L | 82B | 86 |
| D | 86 | 87 | T | 83 | 87 | V | 84 | 87 | T | 83 | 87 |
| T | 87 | 88 | A | 84 | 88 | A | 85 | 88 | A | 84 | 88 |
| A | 88 | 89 | A | 85 | 89 | D | 86 | 89 | A | 85 | 89 |
| T | 89 | 90 | D | 86 | 90 | T | 87 | 90 | D | 86 | 90 |
| Y | 90 | 91 | T | 87 | 91 | A | 88 | 91 | T | 87 | 91 |
| F | 91 | 92 | A | 88 | 92 | T | 89 | 92 | A | 88 | 92 |
| C | 92 | 93 | T | 89 | 93 | Y | 90 | 93 | T | 89 | 93 |
| A | 93 | 94 | Y | 90 | 94 | F | 91 | 94 | Y | 90 | 94 |
| R | 94 | 95 | F | 91 | 95 | C | 92 | 95 | F | 91 | 95 |
| D | 95 | 96 | C | 92 | 96 | A | 93 | 96 | C | 92 | 96 |
| P | 96 | 97 | A | 93 | 97 | R | 94 | 97 | A | 93 | 97 |
| D | 97 | 98 | R | 94 | 98 | D | 95 | 98 | R | 94 | 98 |
| Y | 98 | 99 | D | 95 | 99 | G | 96 | 99 | E | 95 | 99 |
| S | 99 | 100 | G | 96 | 100 | S | 97 | 100 | S | 96 | 100 |
| S | 100 | 101 | S | 97 | 101 | D | 98 | 101 | V | 97 | 101 |
| G | 100A | 102 | D | 98 | 102 | S | 99 | 102 | G | 98 | 102 |
| T | 100B | 103 | S | 99 | 103 | A | 100 | 103 | S | 99 | 103 |
| A | 101 | 104 | G | 100 | 104 | D | 100A | 104 | A | 100 | 104 |
| L | 102 | 105 | D | 100A | 105 | Y | 100B | 105 | Y | 100A | 105 |
| W | 103 | 106 | Y | 100B | 106 | R | 100C | 106 | Y | 100B | 106 |
| G | 104 | 107 | R | 100C | 107 | L | 100D | 107 | T | 100C | 107 |
| P | 105 | 108 | L | 100D | 108 | D | 101 | 108 | F | 100D | 108 |
| G | 106 | 109 | D | 101 | 109 | L | 102 | 109 | D | 101 | 109 |
| T | 107 | 110 | L | 102 | 110 | W | 103 | 110 | L | 102 | 110 |
| L | 108 | 111 | W | 103 | 111 | G | 104 | 111 | W | 103 | 111 |
| V | 109 | 112 | G | 104 | 112 | Q | 105 | 112 | G | 104 | 112 |
| T | 110 | 113 | Q | 105 | 113 | G | 106 | 113 | P | 105 | 113 |
| V | 111 | 114 | G | 106 | 114 | T | 107 | 114 | G | 106 | 114 |
| S | 112 | 115 | T | 107 | 115 | L | 108 | 115 | T | 107 | 115 |
| S | 113 | 116 | L | 108 | 116 | V | 109 | 116 | L | 108 | 116 |
| | | | V | 109 | 117 | T | 110 | 117 | V | 109 | 117 |
| | | | T | 110 | 118 | V | 111 | 118 | T | 110 | 118 |
| | | | V | 111 | 119 | S | 112 | 119 | V | 111 | 119 |
| | | | S | 112 | 120 | S | 113 | 120 | S | 112 | 120 |
| | | | S | 113 | 121 | | | | S | 113 | 121 |

**Table S9.** Chothia and sequential numbering systems for the aεFab $V_L$ domains.

| aεFab67 | | | aεFab81 | | | aεFab92 | | | aεFab98 | | |
|---|---|---|---|---|---|---|---|---|---|---|---|
| **Residue** | **Chothia** | **Sequential** | **Residue** | **Chothia** | **Sequential** | **Residue** | **Chothia** | **Sequential** | **Residue** | **Chothia** | **Sequential** |
| A | 1 | 1 | A | 1 | 1 | A | 1 | 1 | A | 1 | 1 |
| L | 2 | 2 | Y | 2 | 2 | Y | 2 | 2 | Y | 2 | 2 |
| V | 3 | 3 | D | 3 | 3 | D | 3 | 3 | D | 3 | 3 |
| M | 4 | 4 | M | 4 | 4 | M | 4 | 4 | M | 4 | 4 |
| T | 5 | 5 | T | 5 | 5 | T | 5 | 5 | T | 5 | 5 |
| Q | 6 | 6 | Q | 6 | 6 | Q | 6 | 6 | Q | 6 | 6 |
| T | 7 | 7 | T | 7 | 7 | T | 7 | 7 | T | 7 | 7 |
| P | 8 | 8 | P | 8 | 8 | P | 8 | 8 | P | 8 | 8 |
| S | 9 | 9 | A | 9 | 9 | A | 9 | 9 | A | 9 | 9 |
| S | 10 | 10 | S | 10 | 10 | S | 10 | 10 | S | 10 | 10 |
| V | 11 | 11 | V | 11 | 11 | V | 11 | 11 | V | 11 | 11 |
| S | 12 | 12 | E | 12 | 12 | E | 12 | 12 | E | 12 | 12 |
| A | 13 | 13 | V | 13 | 13 | V | 13 | 13 | V | 13 | 13 |
| A | 14 | 14 | A | 14 | 14 | G | 14 | 14 | A | 14 | 14 |
| V | 15 | 15 | V | 15 | 15 | V | 15 | 15 | V | 15 | 15 |
| G | 16 | 16 | G | 16 | 16 | G | 16 | 16 | G | 16 | 16 |
| G | 17 | 17 | G | 17 | 17 | G | 17 | 17 | G | 17 | 17 |
| T | 18 | 18 | T | 18 | 18 | T | 18 | 18 | T | 18 | 18 |
| V | 19 | 19 | V | 19 | 19 | V | 19 | 19 | V | 19 | 19 |
| T | 20 | 20 | T | 20 | 20 | T | 20 | 20 | T | 20 | 20 |
| I | 21 | 21 | I | 21 | 21 | I | 21 | 21 | I | 21 | 21 |
| K | 22 | 22 | K | 22 | 22 | K | 22 | 22 | K | 22 | 22 |
| C | 23 | 23 | C | 23 | 23 | C | 23 | 23 | C | 23 | 23 |
| Q | 24 | 24 | Q | 24 | 24 | Q | 24 | 24 | Q | 24 | 24 |
| A | 25 | 25 | A | 25 | 25 | A | 25 | 25 | A | 25 | 25 |
| S | 26 | 26 | S | 26 | 26 | S | 26 | 26 | S | 26 | 26 |
| Q | 27 | 27 | Q | 27 | 27 | Q | 27 | 27 | Q | 27 | 27 |
| S | 28 | 28 | S | 28 | 28 | N | 28 | 28 | S | 28 | 28 |
| I | 29 | 29 | I | 29 | 29 | I | 29 | 29 | I | 29 | 29 |
| G | 30 | 30 | S | 30 | 30 | Y | 30 | 30 | S | 30 | 30 |
| S | 31 | 31 | S | 31 | 31 | S | 31 | 31 | N | 31 | 31 |
| Y | 32 | 32 | Y | 32 | 32 | N | 32 | 32 | A | 32 | 32 |
| L | 33 | 33 | L | 33 | 33 | L | 33 | 33 | L | 33 | 33 |
| N | 34 | 34 | A | 34 | 34 | A | 34 | 34 | S | 34 | 34 |
| W | 35 | 35 | W | 35 | 35 | W | 35 | 35 | W | 35 | 35 |
| Y | 36 | 36 | Y | 36 | 36 | Y | 36 | 36 | Y | 36 | 36 |
| Q | 37 | 37 | Q | 37 | 37 | Q | 37 | 37 | Q | 37 | 37 |
| Q | 38 | 38 | Q | 38 | 38 | Q | 38 | 38 | Q | 38 | 38 |
| K | 39 | 39 | K | 39 | 39 | K | 39 | 39 | K | 39 | 39 |
| P | 40 | 40 | P | 40 | 40 | P | 40 | 40 | P | 40 | 40 |
| G | 41 | 41 | G | 41 | 41 | G | 41 | 41 | G | 41 | 41 |
| Q | 42 | 42 | Q | 42 | 42 | Q | 42 | 42 | Q | 42 | 42 |
| P | 43 | 43 | P | 43 | 43 | R | 43 | 43 | R | 43 | 43 |
| P | 44 | 44 | P | 44 | 44 | P | 44 | 44 | P | 44 | 44 |
| K | 45 | 45 | K | 45 | 45 | K | 45 | 45 | K | 45 | 45 |
| L | 46 | 46 | L | 46 | 46 | L | 46 | 46 | L | 46 | 46 |
| L | 47 | 47 | L | 47 | 47 | L | 47 | 47 | L | 47 | 47 |
| I | 48 | 48 | I | 48 | 48 | I | 48 | 48 | I | 48 | 48 |
| Y | 49 | 49 | Y | 49 | 49 | Y | 49 | 49 | Y | 49 | 49 |
| K | 50 | 50 | D | 50 | 50 | G | 50 | 50 | D | 50 | 50 |
| A | 51 | 51 | A | 51 | 51 | A | 51 | 51 | A | 51 | 51 |
| S | 52 | 52 | S | 52 | 52 | S | 52 | 52 | S | 52 | 52 |
| T | 53 | 53 | T | 53 | 53 | N | 53 | 53 | K | 53 | 53 |
| L | 54 | 54 | L | 54 | 54 | L | 54 | 54 | M | 54 | 54 |
| A | 55 | 55 | A | 55 | 55 | E | 55 | 55 | A | 55 | 55 |
| S | 56 | 56 | S | 56 | 56 | S | 56 | 56 | S | 56 | 56 |
| G | 57 | 57 | G | 57 | 57 | G | 57 | 57 | G | 57 | 57 |
| V | 58 | 58 | V | 58 | 58 | V | 58 | 58 | V | 58 | 58 |
| S | 59 | 59 | P | 59 | 59 | P | 59 | 59 | P | 59 | 59 |
| S | 60 | 60 | S | 60 | 60 | S | 60 | 60 | S | 60 | 60 |
| R | 61 | 61 | R | 61 | 61 | R | 61 | 61 | R | 61 | 61 |
| F | 62 | 62 | F | 62 | 62 | F | 62 | 62 | F | 62 | 62 |
| R | 63 | 63 | K | 63 | 63 | K | 63 | 63 | K | 63 | 63 |
| G | 64 | 64 | G | 64 | 64 | G | 64 | 64 | G | 64 | 64 |
| S | 65 | 65 | S | 65 | 65 | S | 65 | 65 | S | 65 | 65 |
| G | 66 | 66 | G | 66 | 66 | G | 66 | 66 | G | 66 | 66 |
| S | 67 | 67 | Y | 67 | 67 | S | 67 | 67 | S | 67 | 67 |

| | | | | | | | | | | | |
|---|---|---|---|---|---|---|---|---|---|---|---|
| G | 68 | 68 | G | 68 | 68 | G | 68 | 68 | G | 68 | 68 |
| T | 69 | 69 | T | 69 | 69 | T | 69 | 69 | T | 69 | 69 |
| E | 70 | 70 | E | 70 | 70 | E | 70 | 70 | E | 70 | 70 |
| F | 71 | 71 | F | 71 | 71 | Y | 71 | 71 | F | 71 | 71 |
| T | 72 | 72 | T | 72 | 72 | T | 72 | 72 | T | 72 | 72 |
| L | 73 | 73 | L | 73 | 73 | L | 73 | 73 | L | 73 | 73 |
| T | 74 | 74 | T | 74 | 74 | T | 74 | 74 | T | 74 | 74 |
| I | 75 | 75 | I | 75 | 75 | I | 75 | 75 | I | 75 | 75 |
| S | 76 | 76 | S | 76 | 76 | S | 76 | 76 | S | 76 | 76 |
| D | 77 | 77 | G | 77 | 77 | G | 77 | 77 | D | 77 | 77 |
| L | 78 | 78 | V | 78 | 78 | V | 78 | 78 | L | 78 | 78 |
| E | 79 | 79 | E | 79 | 79 | E | 79 | 79 | E | 79 | 79 |
| C | 80 | 80 | C | 80 | 80 | C | 80 | 80 | C | 80 | 80 |
| A | 81 | 81 | A | 81 | 81 | A | 81 | 81 | D | 81 | 81 |
| D | 82 | 82 | D | 82 | 82 | D | 82 | 82 | D | 82 | 82 |
| A | 83 | 83 | A | 83 | 83 | A | 83 | 83 | A | 83 | 83 |
| A | 84 | 84 | T | 84 | 84 | A | 84 | 84 | A | 84 | 84 |
| T | 85 | 85 | T | 85 | 85 | T | 85 | 85 | T | 85 | 85 |
| Y | 86 | 86 | Y | 86 | 86 | Y | 86 | 86 | Y | 86 | 86 |
| Y | 87 | 87 | Y | 87 | 87 | Y | 87 | 87 | Y | 87 | 87 |
| C | 88 | 88 | C | 88 | 88 | C | 88 | 88 | C | 88 | 88 |
| Q | 89 | 89 | Q | 89 | 89 | Q | 89 | 89 | Q | 89 | 89 |
| Q | 90 | 90 | Q | 90 | 90 | Q | 90 | 90 | Q | 90 | 90 |
| D | 91 | 91 | G | 91 | 91 | G | 91 | 91 | G | 91 | 91 |
| T | 92 | 92 | I | 92 | 92 | A | 92 | 92 | Y | 92 | 92 |
| A | 93 | 93 | T | 93 | 93 | T | 93 | 93 | S | 93 | 93 |
| V | 94 | 94 | I | 94 | 94 | I | 94 | 94 | R | 94 | 94 |
| I | 95 | 95 | S | 95 | 95 | S | 95 | 95 | S | 95 | 95 |
| E | 95A | 96 | G | 95A | 96 | G | 95A | 96 | G | 95A | 96 |
| I | 95B | 97 | V | 95B | 97 | V | 95B | 97 | V | 95B | 97 |
| E | 95C | 98 | E | 95C | 98 | E | 95C | 98 | D | 95C | 98 |
| N | 96 | 99 | N | 96 | 99 | N | 96 | 99 | S | 96 | 99 |
| T | 97 | 100 | V | 97 | 100 | V | 97 | 100 | T | 97 | 100 |
| F | 98 | 101 | F | 98 | 101 | F | 98 | 101 | F | 98 | 101 |
| G | 99 | 102 | G | 99 | 102 | G | 99 | 102 | G | 99 | 102 |
| G | 100 | 103 | G | 100 | 103 | G | 100 | 103 | G | 100 | 103 |
| G | 101 | 104 | G | 101 | 104 | G | 101 | 104 | G | 101 | 104 |
| T | 102 | 105 | T | 102 | 105 | T | 102 | 105 | T | 102 | 105 |
| E | 103 | 106 | E | 103 | 106 | E | 103 | 106 | K | 103 | 106 |
| V | 104 | 107 | V | 104 | 107 | V | 104 | 107 | V | 104 | 107 |
| V | 105 | 108 | V | 105 | 108 | V | 105 | 108 | V | 105 | 108 |
| V | 106 | 109 | V | 106 | 109 | V | 106 | 109 | V | 106 | 109 |
| K | 107 | 110 | E | 107 | 110 | K | 107 | 110 | K | 107 | 110 |
| R | 108 | 111 | R | 108 | 111 | R | 108 | 111 | R | 108 | 111 |
| T | 109 | 112 | T | 109 | 112 | T | 109 | 112 | T | 109 | 112 |
| P | 110 | 113 | P | 110 | 113 | P | 110 | 113 | P | 110 | 113 |

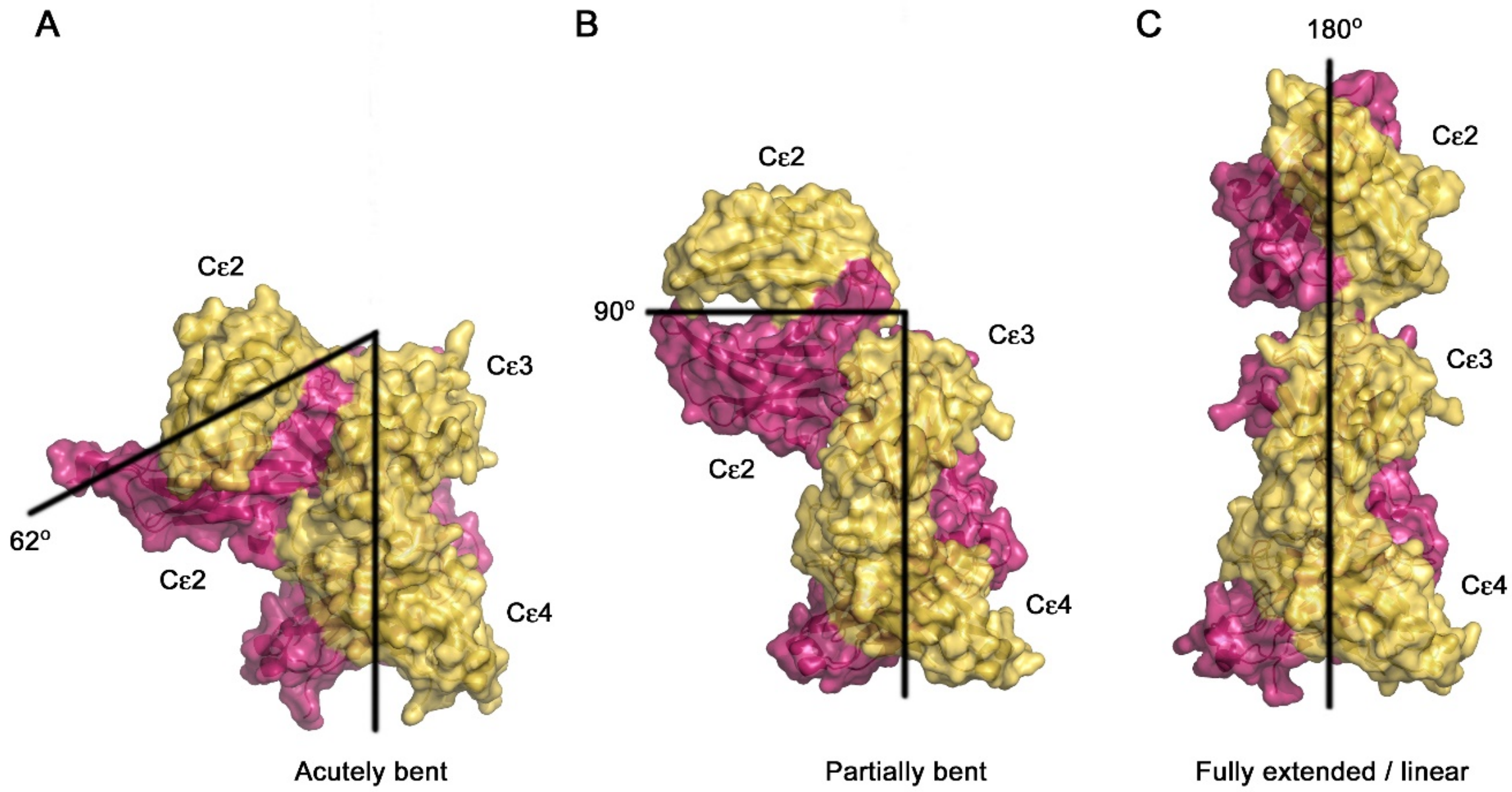


**Figure S1.** Bend angles in IgE-Fc. The bend in IgE-Fc is described by the angle between the local two-fold axes of the $(C\varepsilon2)_2$ and $(C\varepsilon4)_2$ domain pairs. (*A*) Unbound IgE-Fc is acutely bent, with a bend angle of 62° (19). (*B*) IgE-Fc is partially bent when in complex with an omalizumab-derived Fab, with a bend angle of 90° (20). (*C*) IgE-Fc adopts a fully extended, linear conformation in the aεFab complex (11). For clarity, the omalizumab-derived Fab and aεFab are not shown in panels (*B*) and (*C*), respectively. IgE-Fc chains A and B are colored yellow and pink, respectively. For consistency, the chain assignments in IgE-Fc are the same as those in the crystal structure of unbound IgE-Fc (19).

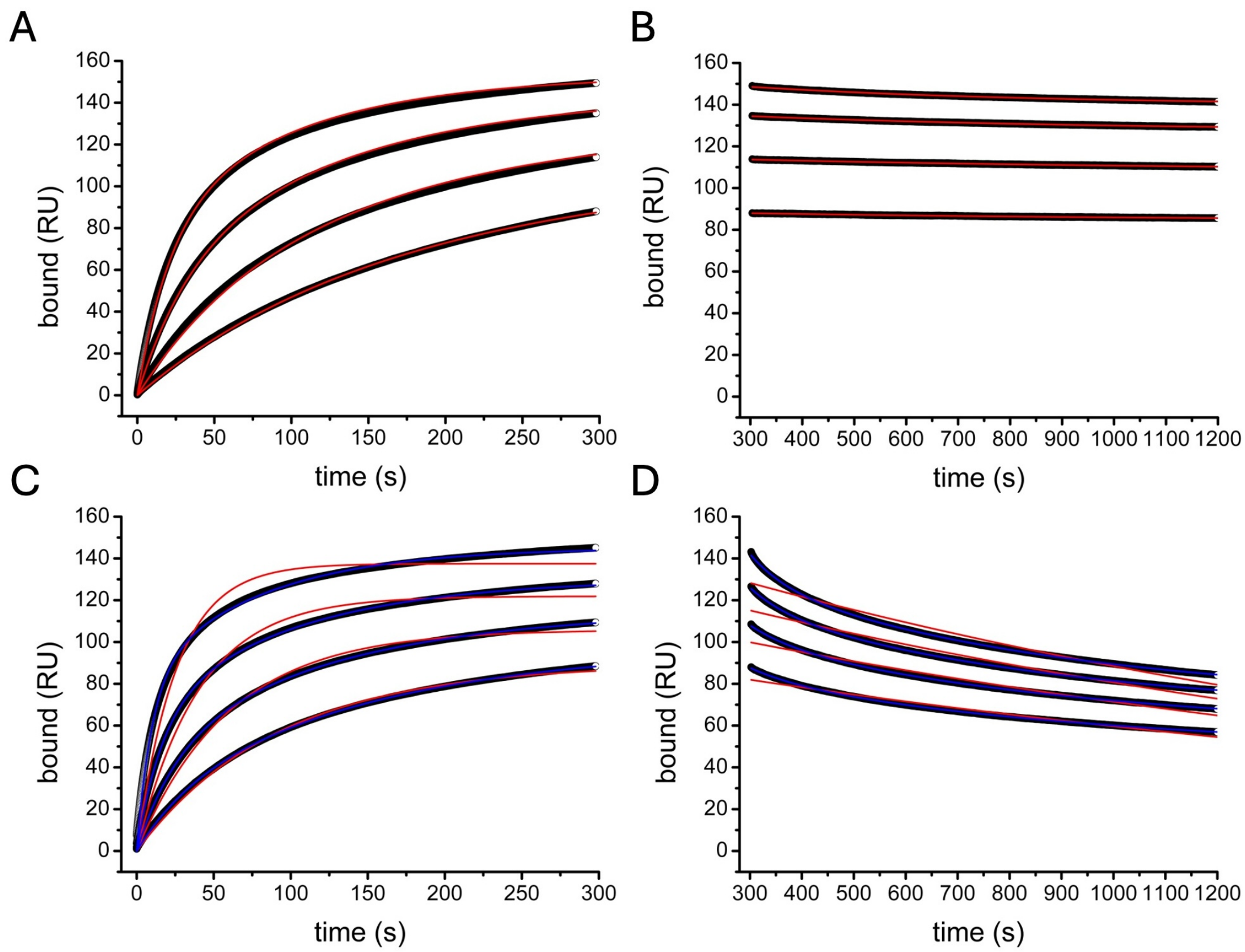


**Figure S2.** Examples of the quality of the kinetic fits for direct SPR binding experiments. (A) association phase and (B) dissociation phase fits for IgE-Fc binding to immobilized aεFab92. Binding data are shown as open black circles and monophasic fits are shown as red lines. (C) association phase and (D) dissociation phase fits for IgE-Fc binding to immobilized aεFab98. Binding data are shown as open black circles, monophasic fits are shown as red lines, and biphasic fits are shown as blue lines. IgE-Fc binding to aεFab92 is well described by a single on-rate constant and a single off-rate constant. IgE-Fc binding to aεFab98 is poorly fit by monophasic fits, and clearly shows a second binding component. See Table S1 for binding rates and affinities.

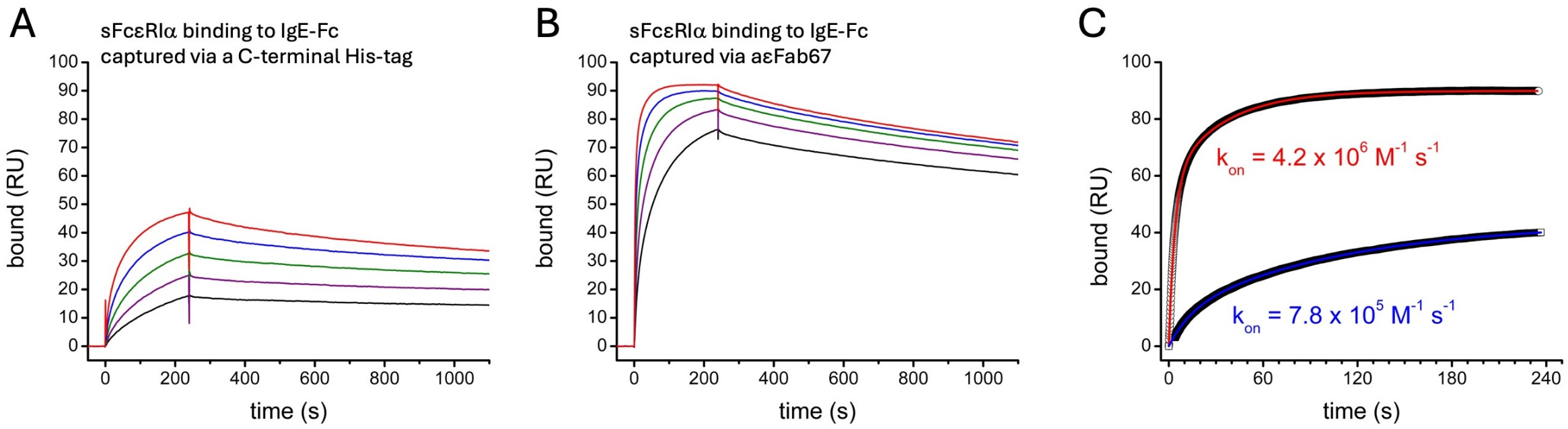


**Figure S3.** A comparison of the binding of sFcεRIα to IgE-Fc versus the IgE-Fc/aεFab67 complex. (*A*) IgE-Fc was captured by a C-terminal His-tag and sFcεRIα was bound at concentrations of 1 nM (black), 2 nM (purple), 4 nM (green), 8 nM (blue) and 16 nM (red). (*B*) IgE-Fc was captured by binding to aεFab67 and sFcεRIα was bound for the same concentration series. (*C*) Marked differences were seen for the association rates for the aεFab67-captured IgE-Fc interaction with sFcεRIα. The side-by-side fits of the association rate constants are shown for the 8 nM concentration. The association fit and the association rate constant for sFcεRIα binding to IgE-Fc is shown in blue; and the fit and rate constant for sFcεRIα binding to the IgE-Fc/aεFab67 complex is shown in red.

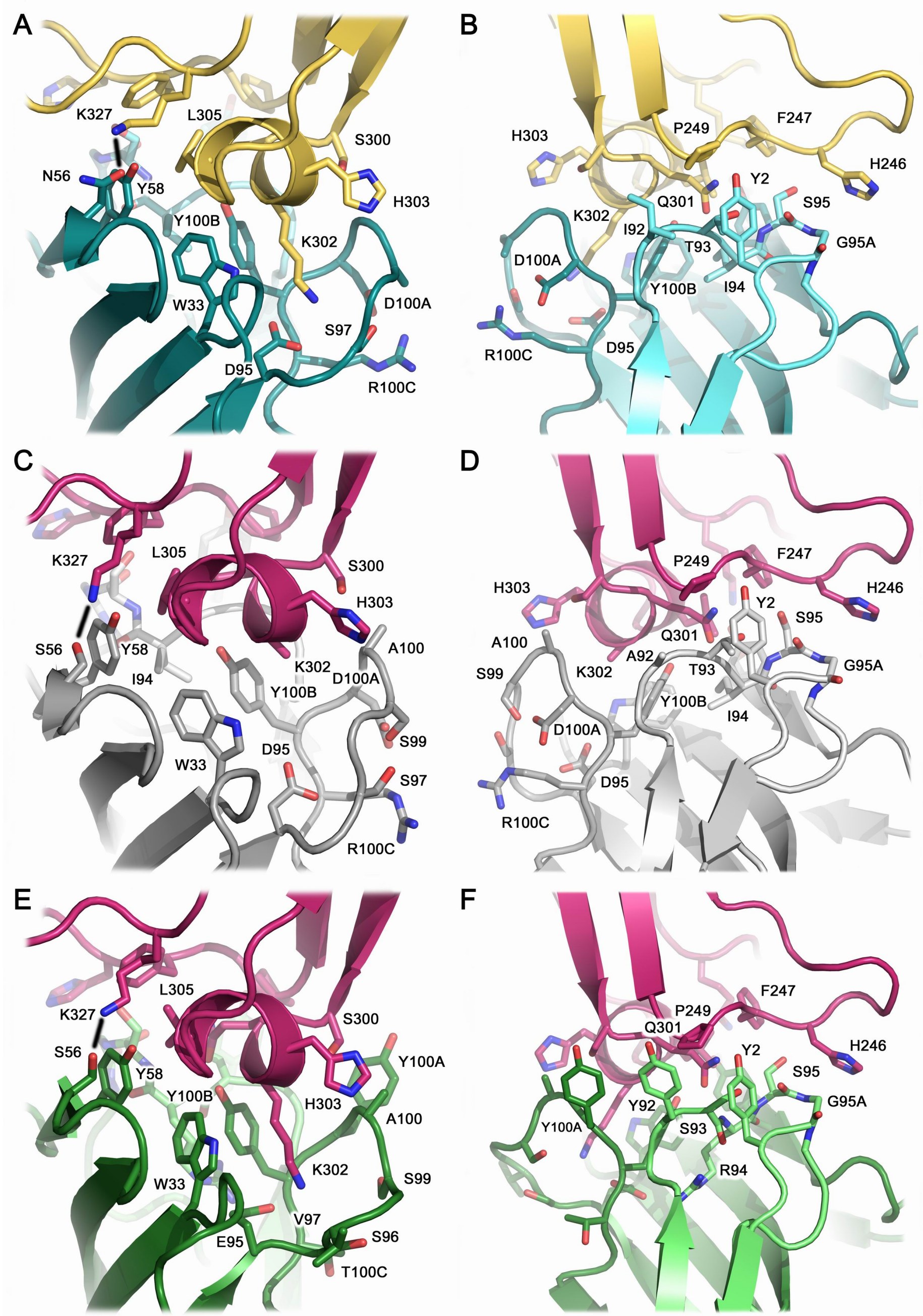


**Figure S4.** Interaction between aεFab81, aεFab92 and aεFab98 and the common epitope on the Cε2 domain. (*A*) Interface between the aεFab81 heavy chain (dark cyan) and the Cε2 domain in chain A (yellow). (*B*) Interface between the aεFab81 light chain (pale cyan) and the Cε2 domain in chain A (yellow). (*C*) Interface between the aεFab92 heavy chain (dark gray) and the Cε2 domain in chain B (pink). (*D*) Interface between the aεFab92 light chain (light gray) and the Cε2 domain in chain B (pink). (*E*) Interface between the aεFab98 heavy chain (dark green) and the Cε2 domain in chain B (pink). (*F*) Interface between the aεFab98 light chain (pale green) and the Cε2 domain in chain B (pink). In panels *A, C* and *E,* hydrogen bonds are depicted by black lines. aεFab residues are numbered according to the Chothia numbering system.

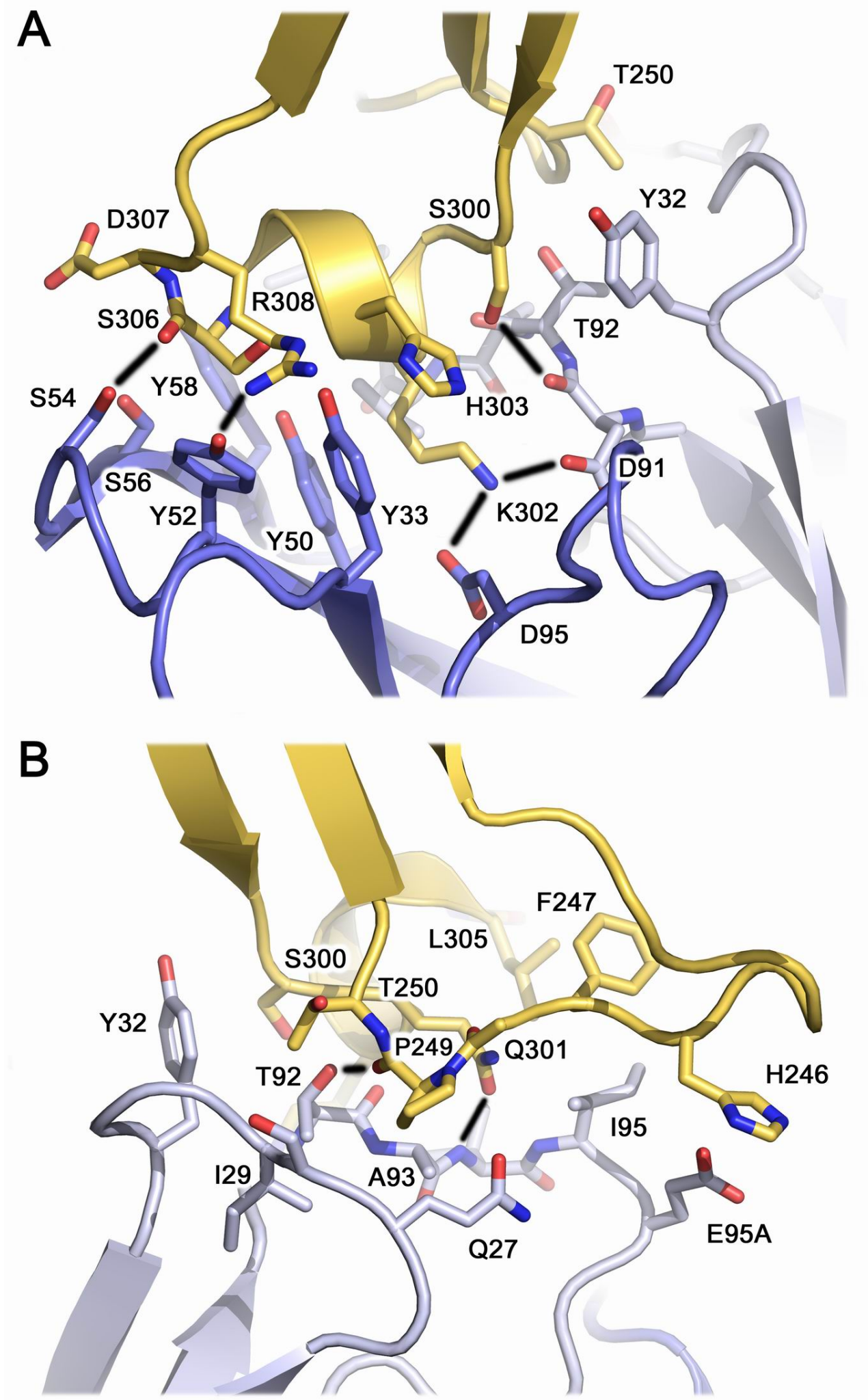


**Figure S5.** Interaction between aεFab67[A] and the common epitope on the Cε2 domain. (*A*) Interface between the aεFab67[A] heavy chain (dark blue) and light chain (light blue) and the Cε2 domain in chain A (yellow). (*B*) Interface between the aεFab67[A] light chain (light blue) and the Cε2 domain in chain A (yellow). In panels *A* and *B,* hydrogen bonds are depicted by black lines. aεFab67 residues are numbered according to the Chothia numbering system.

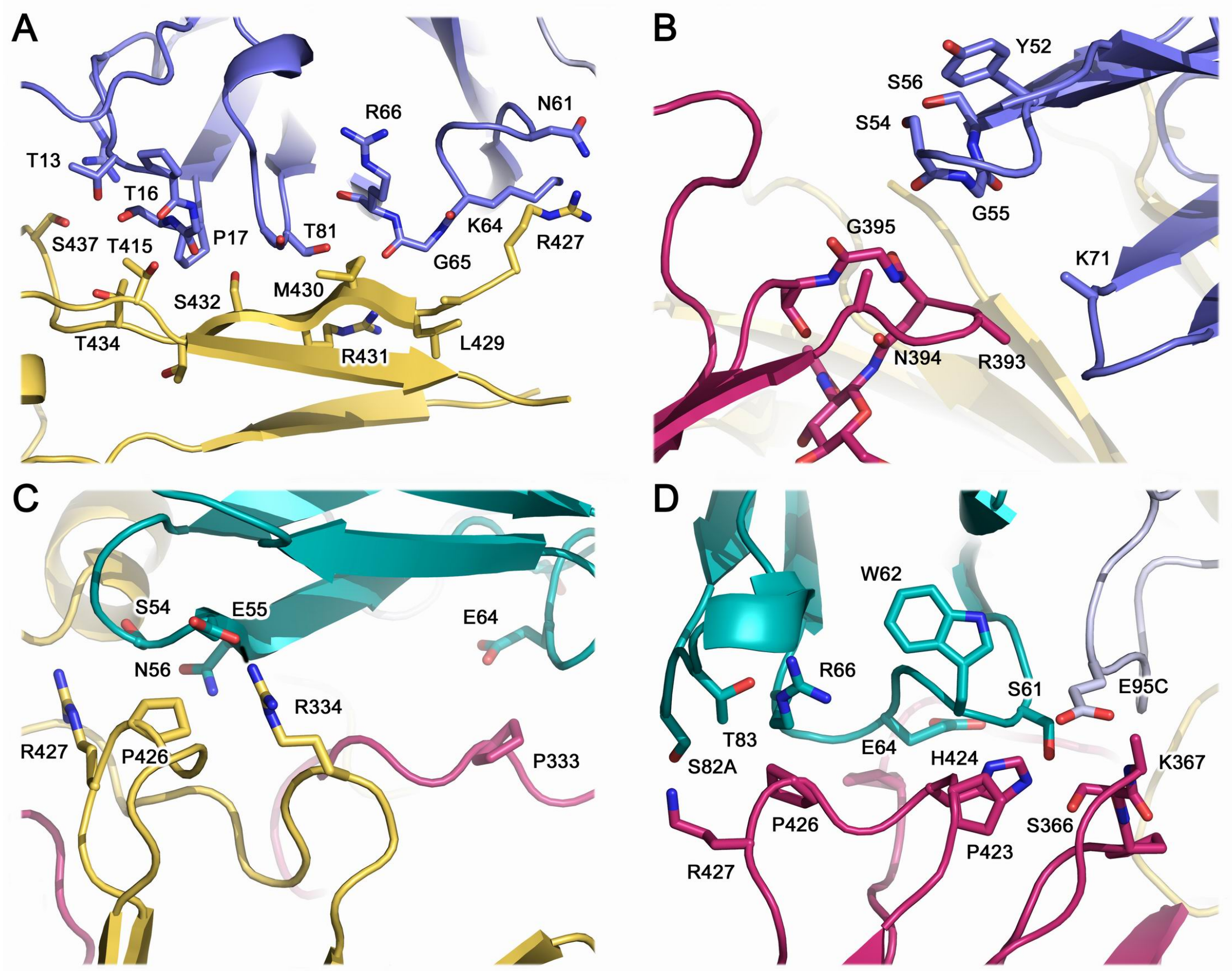


**Figure S6.** Interfaces between aεFab67 and IgE-Fc and aεFab81 and IgE-Fc. (*A*) Interface between the aεFab67$^{B}$ $V_H$ domain (dark blue) and IgE-Fc chain A (yellow). Framework region residues contact the Cε3 domain edge β-strand G. (*B*) Interface between the aεFab67$^{B}$ $V_H$ domain (dark blue) and IgE-Fc chain B (pink). Framework region residues contact the Cε3 domain DE loop. The K71 and R393 side chains are partially disordered. (*C*) Interface between the aεFab81 $V_H$ domain (dark cyan) and IgE-Fc. CDRH2 residues contact the linker between the Cε2 and Cε3 domains in chain A (yellow) and framework residues contact the linker (P333) in chain B (pink). CDRH2 residues contact the Cε3 domain FG loop in chain A. The salt bridge between E55 (CDRH2) and R334 (Cε2-Cε3 linker) is depicted by a black line. (*D*) aεFab81 $V_H$ domain framework region residues (dark cyan) contact the Cε3 domain FG loop in chain B (pink). CDRL3 residues (pale cyan) contact the Cε3 domain BC loop in chain B (pink). The K367 and R427 side chains are partially disordered. aεFab residues are numbered according to the Chothia numbering system.

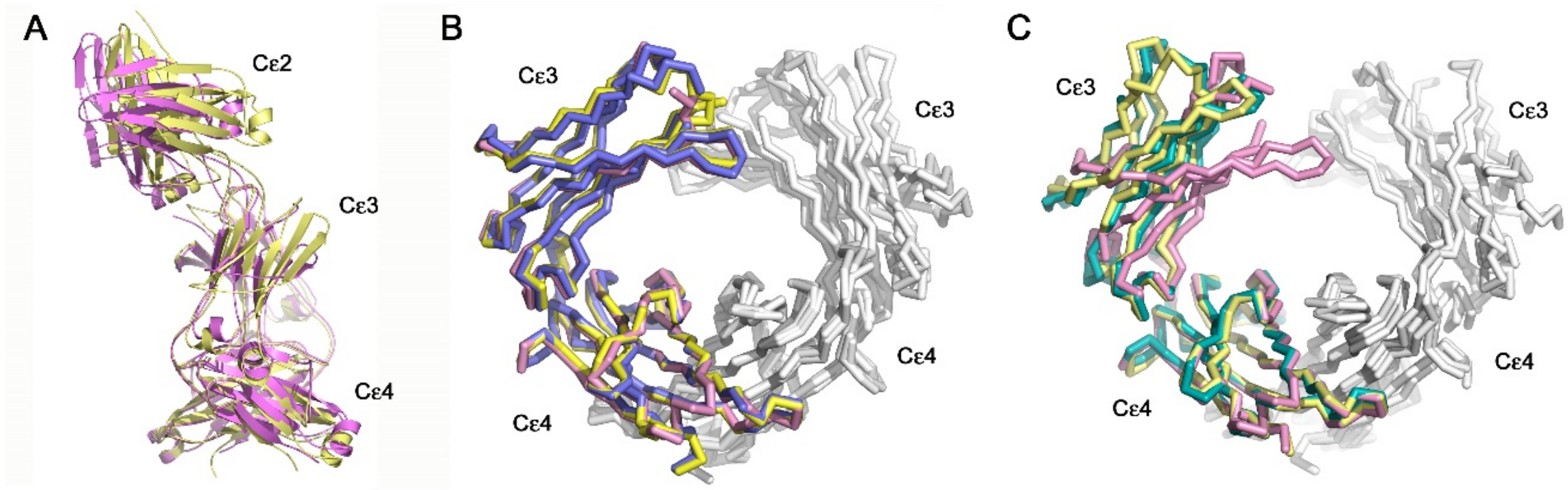


**Figure S7.** Overall structure of IgE-Fc in the aεFab92/IgE-Fc and aεFab98/IgE-Fc complexes. (*A*) The IgE-Fc bend angles are similar in the aεFab92/IgE-Fc (pink) and aεFab98/IgE-Fc (yellow) complexes. (*B*) In the aεFab92/IgE-Fc (pink) and aεFab98/IgE-Fc (yellow) complexes, the Cε3 domain in chain B adopts a closed conformation that is comparable to the closed conformation compatible with CD23 binding (e.g. pdb_00004ezm, blue) (21). Chain A is colored light gray. (*C*) The Cε3 domain in chain A adopts a closed conformation in the aεFab92/IgE-Fc complex (pink), but a markedly open conformation in the aεFab98/IgE-Fc complex (yellow) that is comparable to the open conformation observed in the complex between IgE-Fc and an omalizumab-derived Fab (dark cyan) (20). Chain B is colored light gray.

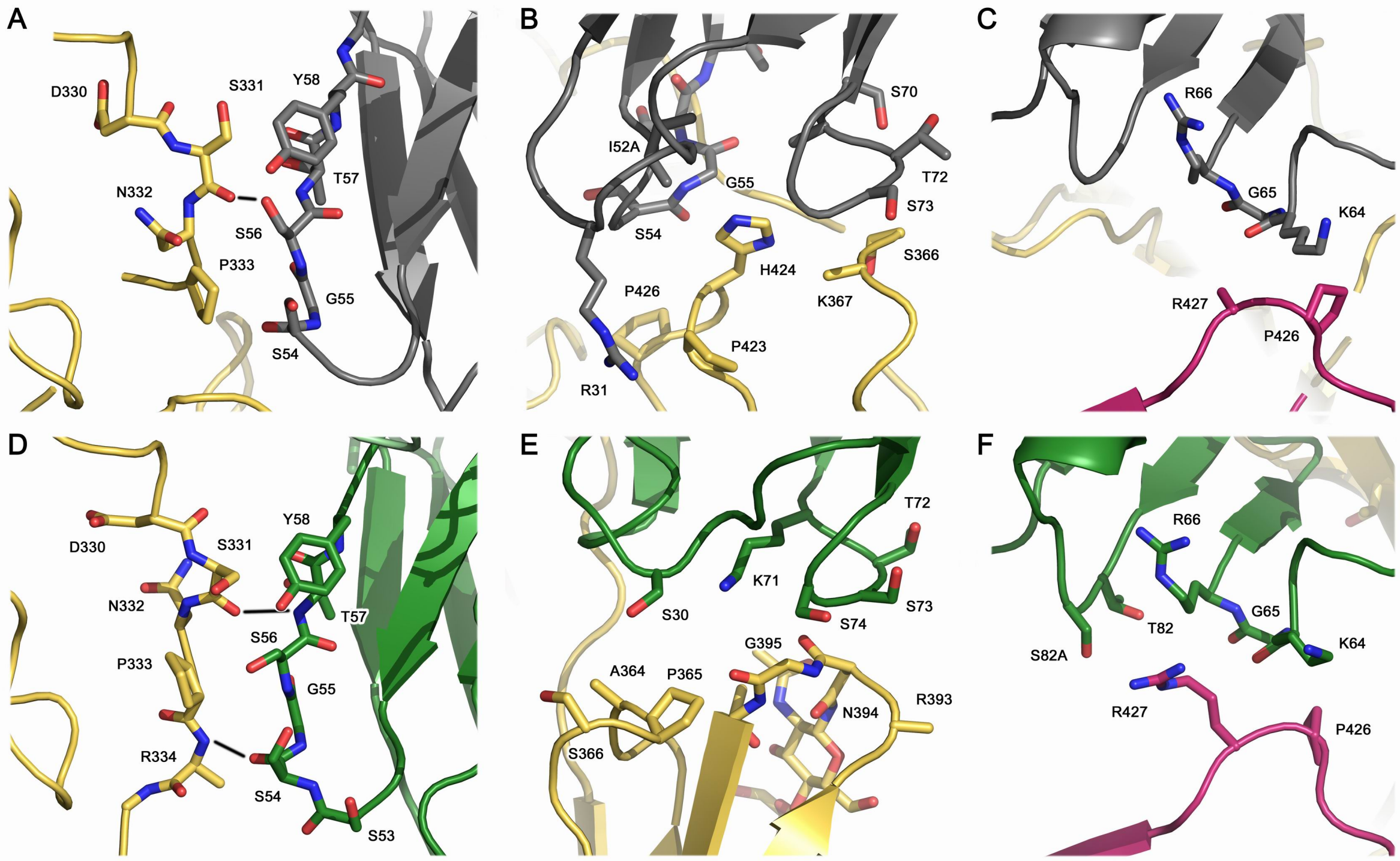


**Figure S8.** Interfaces between aεFab92 and IgE-Fc and aεFab98 and IgE-Fc. (*A*) Interface between CDRH2 residues from the aεFab92 $V_H$ domain (dark gray) and the Cε2-Cε3 domain linker in chain A (yellow). (*B*) aεFab92 framework region residues (dark gray) pack against the Cε3 domain BC loop in chain A (yellow), and CDRH2 residues contact the Cε3 FG loop. The K367 side chain is partially disordered. (*C*) aεFab92 framework region residues (dark gray) contact the Cε3 domain FG loop in chain B (pink). The R427 side chain is partially disordered. (*D*) Interface between CDRH2 residues from the aεFab98 $V_H$ domain (dark green) and the Cε2-Cε3 domain linker in chain A (yellow). The R334 side chain is partially disordered. (*E*) aεFab98 framework region residues (dark green) pack against the Cε3 domain DE loop in chain A (yellow). The R393 side chain is partially disordered. (*F*) aεFab98 framework region residues (dark green) contact the Cε3 domain FG loop in chain B (pink). In panels *A* and *D,* hydrogen bonds are depicted by black lines. aεFab residues are numbered according to the Chothia numbering system.

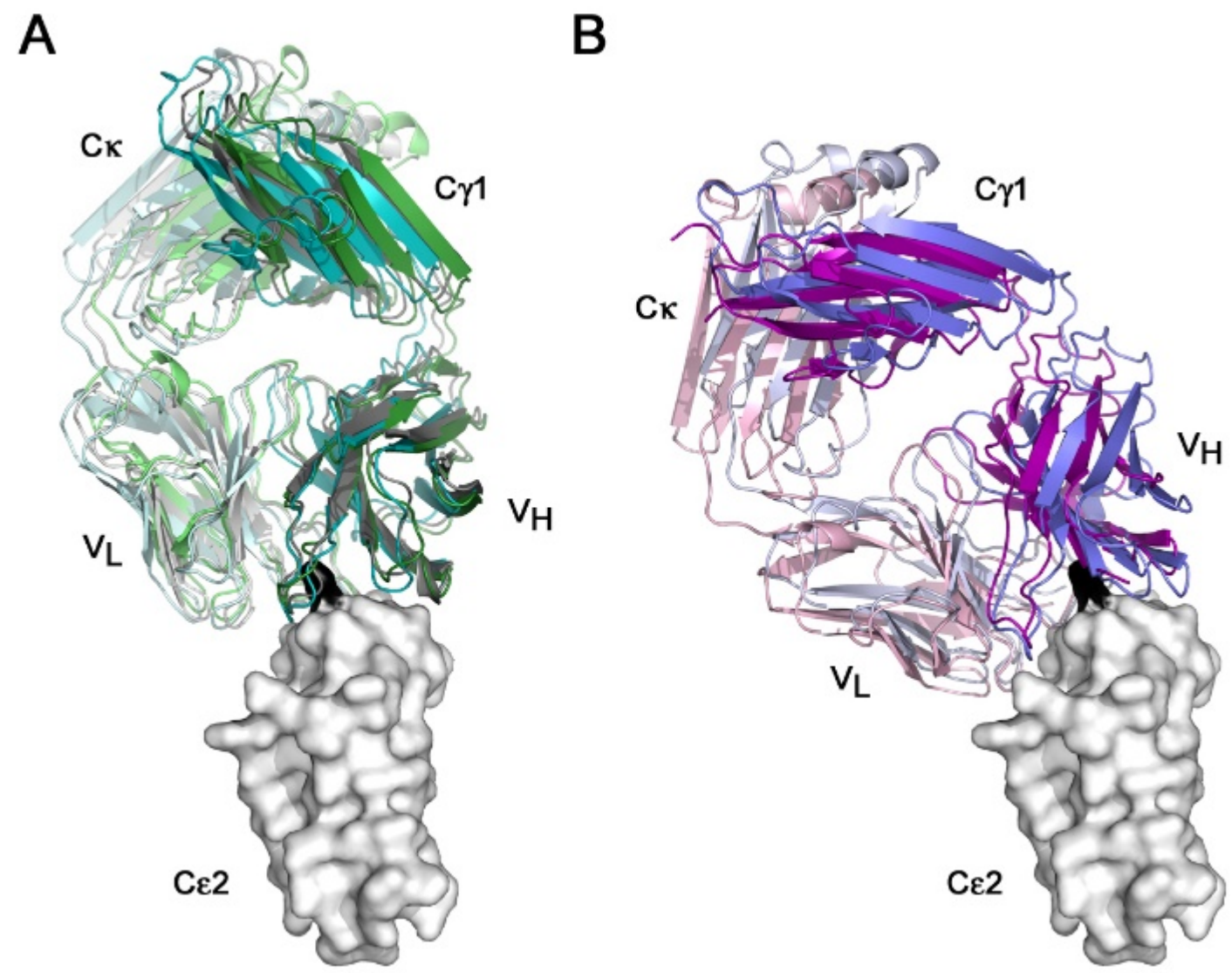


**Figure S9.** Overall orientation of the aεFabs. (A) aεFab81 (dark and pale cyan), aεFab92 (dark and light gray) and aεFab98 (dark and pale green) adopt similar orientations relative to the Cε2 domain. (*B*) aεFab67$^A$ (dark and light blue) and aεFab67$^B$ (dark and light pink) adopt a different orientation compared to the other aεFabs. Structures were superposed on the Cε2 domain. In panels *A* and *B*, the Cε2 domain is colored light gray. K302, which forms part of the common epitope, is colored black.

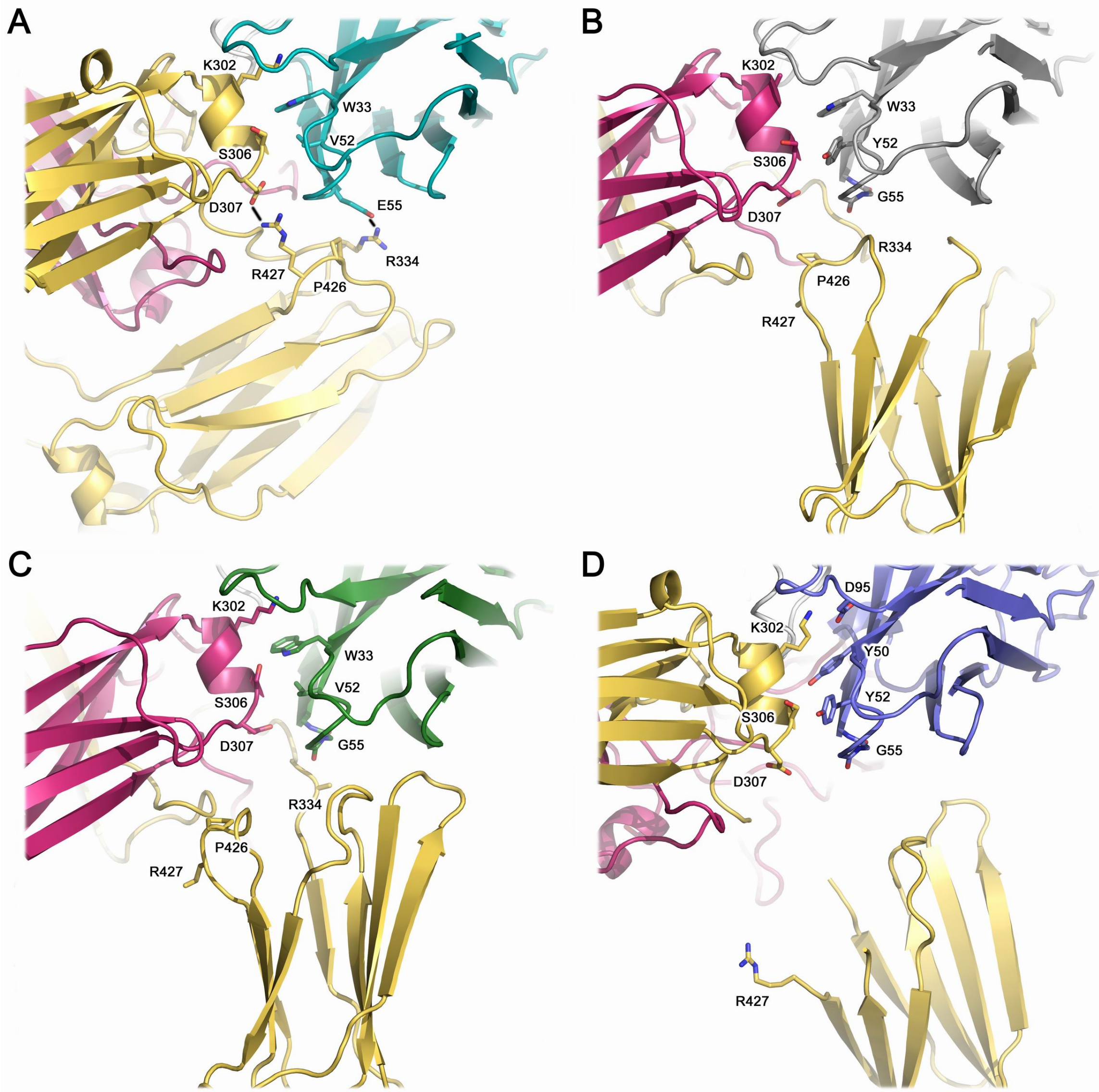


**Figure S10.** Sequence differences in CDRH2. (*A*) In the aεFab81/IgE-Fc complex, E55 (CDRH2) forms a salt bridge with R334 in the Cε2-Cε3 domain linker. IgE-Fc adopts an acutely bent conformation, with the formation of an additional salt bridge between D307 (Cε2) and R427 (Cε3). The salt bridges are depicted by black lines. (*B*) The aεFab92 CDRH2 contains a glycine residue at position 55, which precludes the formation of a salt bridge with R334. IgE-Fc is partially extended, with a bend angle of ~129°. The K302, R334 and R427 side chains are partially disordered. (*C*) The aεFab98 CDRH2 contains a glycine residue at position 55, which precludes the formation of a salt bridge with R334. IgE-Fc is partially extended, with a bend angle of ~132°. The R334 and R427 side chains are partially disordered. (*D*) The aεFab67 CDRH2 contains a glycine residue at position 55, which precludes the formation of a salt bridge with R334. IgE-Fc is partially extended, with a bend angle of ~151°. In this structure, the Cε2-Cε3 domain linker in chain A is partially disordered. In panels *A-D*, IgE-Fc chains A and B are colored yellow and pink, respectively. In each panel, K302 from the common epitope is shown. The Fab carbon atoms are colored as follows: aεFab81, dark cyan; aεFab92, dark gray; aεFab98, dark green; aεFab67, dark blue. aεFab residues are numbered according to the Chothia numbering system.

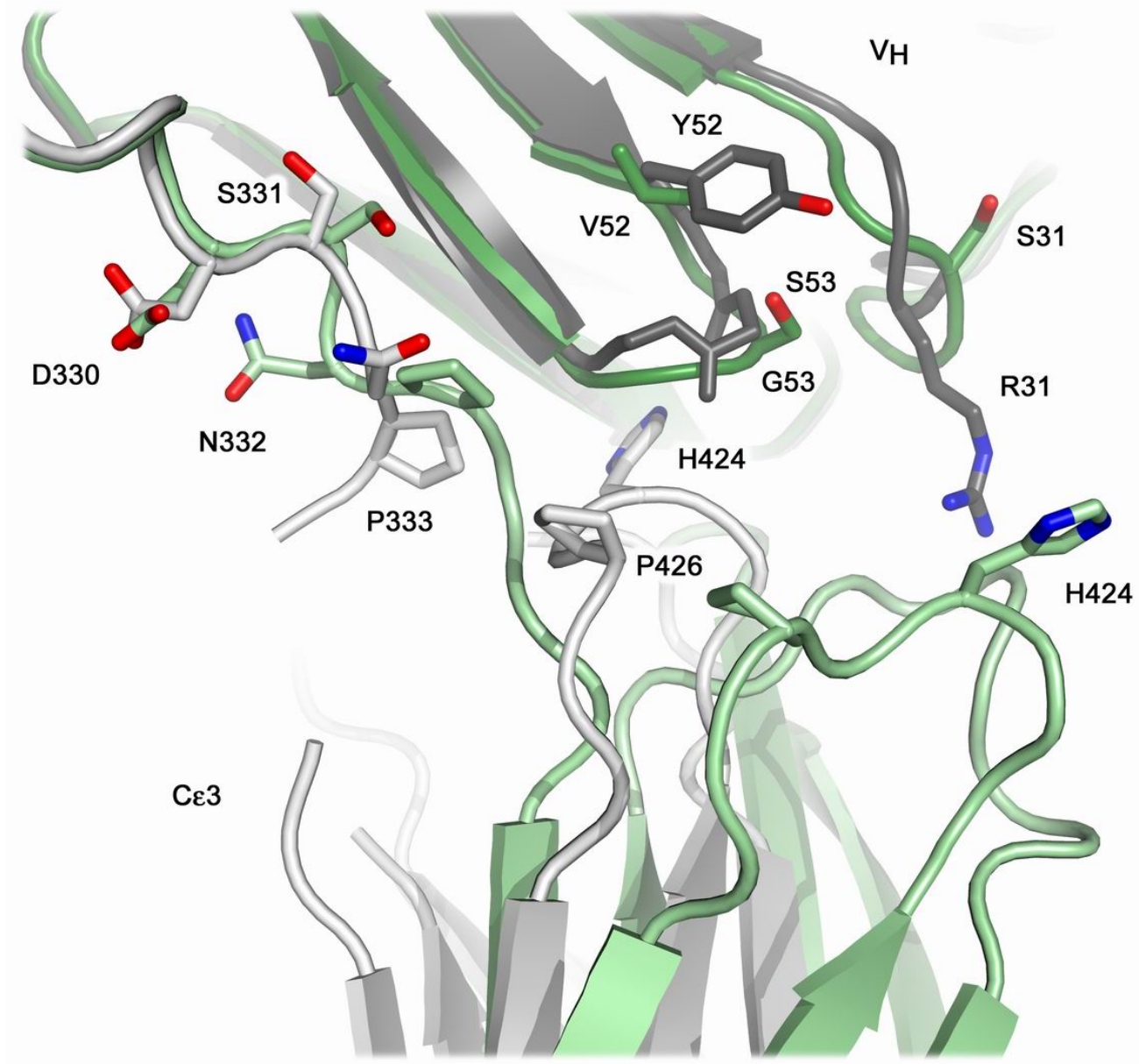


**Figure S11.** Conformational differences between CDRH1 and CDRH2 in the aεFab92 and aεFab98 complexes with IgE-Fc. In both complexes, CDRH2 contacts the Cε2-Cε3 domain linker (residues D330-P333 are shown). CDRH2 also contacts the Cε3 domain in the aεFab92 complex. Substitutions in CDRH2 are associated with a main chain shift, and the conformation of CDRH1 is also affected. The different structures adopted by these CDRs could modulate the conformation of IgE-Fc in solution. The color scheme for carbon atoms is as follows: aεFab92 $V_H$ domain, dark gray; aεFab98 $V_H$ domain, dark green; chain A Cε3 domain in the aεFab92/IgE-Fc complex, light gray; chain A Cε3 domain in the aεFab98/IgE-Fc complex, pale green. aεFab residues are numbered according to the Chothia numbering system.

**Movie S1 (separate file).** Conformation of IgE-Fc in the aεFab98/IgE-Fc and aεFab92/IgE-Fc complexes. The movie shows a surface representation of IgE-Fc. IgE-Fc chains A and B are colored yellow and pink respectively. The movie begins by showing the partially extended structure for IgE-Fc in the aεFab98/IgE-Fc complex. This partially extended conformation is then morphed to the partially extended conformation in the aεFab92/IgE-Fc complex. Transformation between the two conformations involves closure of the Cε3 domain in chain A and rocking of the $(C\varepsilon2)_2$ domain pair towards the Cε3 domain in chain B.

**Movie S2 (separate file).** Rotation of the $(C\varepsilon2)_2$ domain pair in the aεFab67/IgE-Fc complex. The movie shows a surface representation of IgE-Fc. IgE-Fc chains A and B are colored yellow and pink respectively. The movie begins by showing the acutely bent structure for unbound IgE-Fc. The acutely bent conformation is morphed to the partially bent conformation observed in the omalizumab-derived Fab/IgE-Fc complex, and then to the partially extended conformation in the aεFab67/IgE-Fc complex. Transformation from the partially bent to the partially extended conformation involves closure of both Cε3 domains and rocking of the $(C\varepsilon2)_2$ domain pair towards the Cε3 domain in chain B.

**Movie S3 (separate file).** Rotation of the $(C\varepsilon2)_2$ domain pair in the aεFab98/IgE-Fc complex. The movie shows a surface representation of IgE-Fc. IgE-Fc chains A and B are colored yellow and pink respectively. The movie begins by showing the acutely bent structure for unbound IgE-Fc. The acutely bent conformation is morphed to the partially bent conformation observed in the omalizumab-derived Fab/IgE-Fc complex, and then to the partially extended conformation observed in the aεFab98/IgE-Fc complex. Transformation from the partially bent to the partially extended conformation involves closure of the Cε3 domain in chain B and an anti-clockwise rotation of the $(C\varepsilon2)_2$ domain pair.

## SI References


1. S. Tickle *et al.*, A fully automated primary screening system for the discovery of therapeutic antibodies directly from B cells. *J. Biomol. Screen*. 20, 492-497 (2015).
2. D. Lightwood *et al.*, The discovery, engineering and characterisation of a highly potent anti-human IL-13 Fab fragment designed for administration by inhalation. *J. Mol. Biol.* 425, 577–593 (2012).
3. R. J. Young *et al*., Secretion of recombinant human IgE-Fc by mammalian cells and biological activity of glycosylation site mutants. *Protein Eng*. **8**, 193-199 (1995).
4. D. G. Myszyka, Improving biosensor analysis. *Journal of Molecular Recognition* **12**, 279-284 (1999).
5. W. Kabsch, XDS. *Acta Crystallogr. D Biol. Crystallogr*. **66**, 125-132 (2010).
6. G. Winter, xia2: an expert system for macromolecular crystallography data reduction. *J. Appl. Crystallogr.* **43**, 186-190 (2010).
7. J. Agirre *et al*., The CCP4 suite: integrative software for macromolecular crystallography. *Acta Crystallogr. D Struct. Biol*. **79**, 449-461 (2023).
8. P. R. Evans, G. N. Murshudov, How good are my data and what is the resolution? *Acta Crystallogr. D Biol. Crystallogr*. **69**, 1204–1214 (2013).
9. A. J. McCoy *et al*., Phaser crystallographic software. *J. Appl. Crystallogr*. **40**, 658-674 (2007).
10. M. D. Holdom *et al*., Conformational changes in IgE contribute to its uniquely slow dissociation rate from receptor FcεRI. *Nat. Struct. Mol. Biol*. **18**, 571-576 (2011).
11. N. Drinkwater *et al*., Human immunoglobulin E flexes between acutely bent and extended conformations. *Nat. Struct. Mol. Biol.* **21**, 397-404 (2014).
12. D. Liebschner *et al*., Macromolecular structure determination using X-rays, neutrons and electrons: recent developments in Phenix. *Acta Crystallogr. D Struct*. *Biol.* **75**, 861-877 (2019).
13. P. Emsley, B. Lohkamp, W. G. Scott, K. Cowtan, Features and development of Coot. *Acta*

*Crystallogr. D Biol. Crystallogr*. **66**, 486-501 (2010).
14. V. B. Chen *et al*., MolProbity: all-atom structure validation for macromolecular crystallography. *Acta Crystallogr. D Biol. Crystallogr*. **66**, 12-21 (2010).
15. E. Krissinel, K. Henrick, Inference of macromolecular assemblies from crystalline state. *J. Mol. Biol*. **372**, 774-797 (2007).
16. The PyMOL Molecular Graphics System, Version 1.1r1 Schrödinger, LLC.
17. E. F. Pettersen *et al*., UCSF Chimera--a visualization system for exploratory research and analysis. *J. Comput. Chem*. **25**, 1605-1612 (2004).
18. K. R. Abhinandan, A. C. Martin, Analysis and improvements to Kabat and structurally correct numbering of antibody variable domains. *Mol. Immunol*. **45**, 3832-3839 (2008).
19. T. Wan *et al*., The crystal structure of IgE Fc reveals an asymmetrically bent conformation. *Nat. Immunol*. **3**, 681-686 (2002).
20. A. M. Davies *et al*., Allosteric mechanism of action of the therapeutic anti-IgE antibody omalizumab. *J. Biol. Chem*. **292**, 9975-9987 (2017).
21. B. Dhaliwal *et al*., Crystal structure of IgE bound to its B-cell receptor CD23 reveals a mechanism of reciprocal allosteric inhibition with high affinity receptor FcεRI. *Proc. Natl. Acad. Sci U.S.A* **109**, 12686-12691 (2012).
